# Descriptions of five new Oriental *Ptychoptera* Meigen, 1803 (Diptera, Ptychopteridae)

**DOI:** 10.3897/zookeys.1287.183917

**Published:** 2026-07-31

**Authors:** Levente-Péter Kolcsár, Andrew Fasbender, Nikolai Paramonov, Wichai Srisuka, Yuchen Ang

**Affiliations:** 1 Ditrău, Gábor Áron 75, 537090, Romania Lee Kong Chian Natural History Museum, Faculty of Science, National University of Singapore Singapore Singapore; 2 Rhithron Associates Inc., 33 Fort Missoula Road, Missoula, MT 59804, USA S.I. Vavilov Institute for the History of Science and Technology of Russian Academy of Sciences Moscow Russia; 3 S.I. Vavilov Institute for the History of Science and Technology of Russian Academy of Sciences, 14, Baltiyskaya ul., Moscow, 125315, Russia Unaffiliated Ditrău Romania; 4 Entomology Section, Queen Sirikit Botanic Garden, Chiang Mai 50180, Thailand Rhithron Associates Inc. Missoula United States of America; 5 Lee Kong Chian Natural History Museum, Faculty of Science, National University of Singapore, 2 Conservatory Drive, 117377, Singapore, Singapore Entomology Section, Queen Sirikit Botanic Garden Chiang Mai Thailand

**Keywords:** Fold-winged crane flies, identification key, Indo-Malayan realm, male genitalia, new species, phantom craneflies

## Abstract

Five new Oriental species of *Ptychoptera* Meigen, 1803 are herein described and illustrated: *Ptychoptera hantu* Kolcsár & Fasbender, **sp. nov**. from Singapore, representing the first record of the family from the country; *P. phutphi* Kolcsár & Fasbender, **sp. nov**. from western Thailand; *P. pseudosimilis* Fasbender, **sp. nov**. from Arunachal Pradesh, India; *P. vietnamensis* Paramonov, **sp. nov**. from northwestern Vietnam; and *P. srilankaensis* Paramonov, **sp. nov**. from central Sri Lanka. In addition, *P. annandalei* Brunetti, 1918, *P. perbona* Alexander, 1946, and *P. persimilis* Alexander, 1947 are redescribed, with *P. annandalei* newly recorded from Thailand and *P. perbona* from both Thailand and Vietnam. *Ptychoptera cordata* Zhang & Kang, 2021 is synonymized with *P. annandalei* and *P. perbona flaviventris* Alexander, 1946 with *P. perbona*. An updated key for identifying males of the *P. formosensis* species group is presented.

## Introduction

The family Ptychopteridae (Diptera), commonly known as phantom crane flies or fold-winged crane flies, is a relatively small group within the lower Diptera. Adults are characterized by slender bodies and elongated legs, generally resembling crane flies (Tipulomorpha) and primitive crane flies (Tanyderidae). The family diagnosed by the presence of a prehalter, a finger-like lobe at the base of the halter. Larvae are eucephalous, with short, inflated thoracic segments bearing ventral prolegs; abdominal segments are cylindrical, with segments VII–IX modified into a conical anal division that houses an elongate metapneustic respiratory siphon terminating in a pair of spiracular openings and two small, simple anal papillae. Pupae are fusiform with two prominent thoracic respiratory organs, one enlarged and longer than the body; abdomen bearing rows of crenellated tubercles (Fasbender and Courtney 2017; Wiberg-Larsen et al. 2021).

Ptychopteridae are currently classified into four subfamilies: the extinct Proptychopterininae (12 species) and Eoptychopterinae (61 species), and the extant Ptychopterinae (4 fossil and 92 extant species) and Bittacomorphinae (5 fossil and 13 extant species) (Lin and Lukashevich 2006; Podenas 2007; Lukashevich 2008, 2012; Hao et al. 2009; Lukashevich 2011, 2019, 2020; Krzemiński et al. 2012, 2015; Liu et al. 2012; Fasbender 2014; Fasbender and Courtney 2014; Eskov and Lukashevich 2015; Lukashevich and Arillo 2016; Fasbender and Courtney 2017; Szadziewski et al. 2018; Liu and Huang 2020; Lukashevich and Ansorge 2024). The extant species are classified into three genera: *Ptychoptera* (92 species), *Bittacomorpha* (2 species), and *Bittacomorphella* (11 species) (Nakamura and Saigusa 2009; Nakamura 2012; Kang et al. 2012, 2013, 2019, 2022; Paramonov 2013; Fasbender and Courtney 2017; Boardman 2020; Keresztes et al. 2021; Shao and Kang 2021; Zhang and Kang 2021; Dvořák et al. 2023).

*Ptychoptera* is characterized by the following adult characters: antennae with 13 or 14 flagellomeres; scutum with a complete transverse suture; wings with M forked into M1 and M2; fore tibia bearing a simple, spine-like apical ventral spur; Male genitalia are distinguished by the dorsally rotated aedeagus, while female genitalia possess tergite VII concealing a bilobate epigynium and heavily sclerotized, beak-like cerci (Fasbender and Courtney 2017). The genus is widely distributed in the Palaearctic, Nearctic, Afrotropical, and Oriental regions, and is also present in the Neotropical region (Hancock et al. 2006; Fasbender 2014, 2017; Dvořák et al. 2023).

In the last two decades (2005–2025), 28 new *Ptychoptera* species have been described, increasing the total number of known species by approximately 30%. The majority of these new taxa (21 species) were described from the Eastern Palaearctic and Oriental regions (Nakamura and Saigusa 2009; Nakamura 2012; Kang et al. 2013, 2019, 2022; Paramonov 2013; Shao and Kang 2021; Zhang and Kang 2021), raising the previously known 25 species to 46, which represents an 84% increase. These numbers clearly demonstrate both the high diversity of the Asian *Ptychoptera* fauna and the extent to which it remains poorly studied. However, only a single species has been described from Southeast Asia, namely from the Philippines (Paramonov 2013), while the remaining species were described from China (15 species) and Japan (5 species). Species occurring in China can be identified using Yang et al. (2024), Japanese species by using Nakamura and Saigusa (2009) and Nakamura (2012); whereas those from the Malay Archipelago (Philippines and Indonesia) can be identified using Paramonov (2013).

The aim of this study is to describe five new Oriental species: *Ptychoptera hantu* Kolcsár & Fasbender, sp. nov. from Singapore, representing the first record of Ptychopteridae from the country; *Ptychoptera phutphi* Kolcsár & Fasbender, sp. nov. from western Thailand; *Ptychoptera pseudosimilis* Fasbender, sp. nov. from Arunachal Pradesh, India; *Ptychoptera vietnamensis* Paramonov, sp. nov. from northwestern Vietnam; and *Ptychoptera srilankaensis* Paramonov, sp. nov. from central Sri Lanka. Additionally, *Ptychoptera annandalei* Brunetti, 1918, *Ptychoptera perbona* Alexander, 1946, and *Ptychoptera persimilis* Alexander, 1947 are redescribed, and *P. cordata* Zhang & Kang, 2021 is recognized as a junior synonym of *P. annandalei* and *P. perbona flaviventris* Alexander, 1946 of *P. perbona*. *Ptychoptera annandalei* is recorded from Thailand for the first time, and *P. perbona* from both Thailand and Vietnam. We provide an updated identification key for the males of the *Ptychoptera formosensis* species group.

## Materials and methods

**Figure 1.**
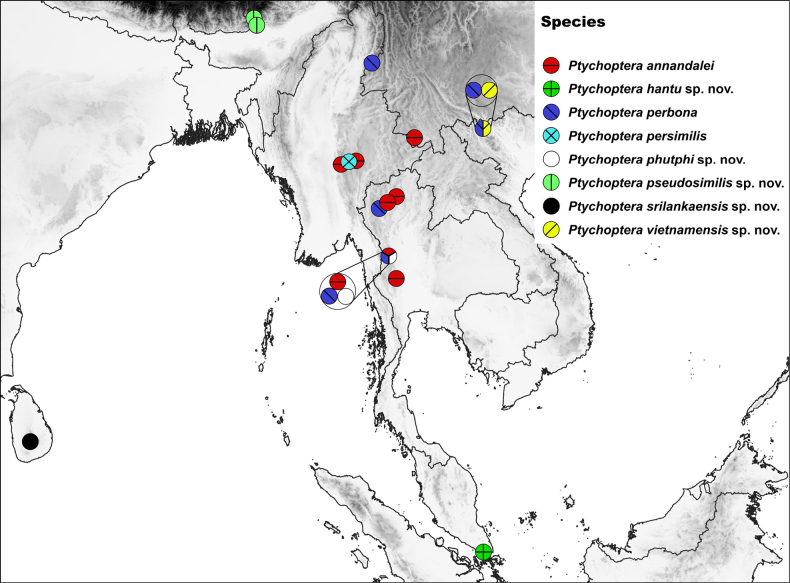
Collection sites.

The specimens were collected using Malaise traps and sweep nets and preserved in 70% ethanol or point-mounted. The approximate collecting location of the studied material is illustrated in Fig. 1. Genitalia were macerated in a 10% solution of potassium hydroxide (KOH), neutralized with 5% acetic acid, and subsequently transferred into plastic tubes containing 50–50% glycerin-ethanol mixture. Photos were taken with a Leica M205C stereomicroscope equipped with a Leica Microsystems DFC5400 camera, which was controlled using the Leica LASX core software (v. 4.3), or with a reflex camera (Canon EOS 7D) paired with a Canon MP-E 65 mm objective for *P. annandalei*, *P. perbona*, *P. hantu* Kolcsár & Fasbender, sp. nov. *P. phutphi* Kolcsár & Fasbender, sp. nov. The photographs of *Ptychoptera srilankaensis* Paramonov, sp. nov. and *P. vietnamensis* Paramonov, sp. nov. were taken with a Canon EOS 800D camera with a MP-E 65mm objective and processed using Helicon Focus 6 software. The macro photographs were taken with a Nikon stereo microscope SMZ25 equipped with objective lens Plan Apo 1.6X and digital camera DS-10. Photomicrographs of *P. pseudosimilis* and *P. persimilis* were produced with a Nikon Eclipse E800 compound microscope equipped with Nomarski DIC using a Nikon DS-Fi1 camera. For aesthetic purposes, the slide photos were edited to remove/repair artifacts such as debris, cracks, and overlapping structures.

Morphological terminology in this study follows Cumming and Wood (2017) and Fasbender (2014, 2017).

Fasbender (2014) proposed the first homology-based terminology for the male genitalia in Ptychopteridae. It was produced as part of a wider investigation into the phylogeny of the extant species of the family, and many of the terms he proposed emphasized explicitly stating the morphological origins of structures. We have shortened several of those terms as they were overly long and cumbersome for practical use. There are two primary lobes of the gonostylus: the basal lobe as described by Fasbender (2014), while his “apical stylus of the gonostylus” *sensu lato* is changed here to “apical lobe”. This better denotes these lobes represent the primordial division of the gonostylus. Further subdivisions of the basal lobe are given a B subscript (i.e., “B_1_, B_2_, B_3_”). For the apical lobe, Fasbender’s “apical stylus of the gonostylus” *sensu stricto* is referred to here as the “Primary Branch,” and his “secondary lobe of the apical stylus of the gonostylus” becomes the “Secondary Branch.” Any further subdivisions of these structures are given a subscript number, “P_1_, P_2_, P_3_” etc. for the primary branch and “S_1_, S_2_, S_3_” for the secondary branch; what Fasbender called “tertiary lobes” are treated as subdivisions of the primary branch. Fasbender’s “membranous window” and “eversible sac” of the hypandrium refer to a weakly sclerotized region that primordially originated in the intersegmental conjunctive between the basal and terminal divisions, we use “hypandrial conjunctive” instead as recently described species have blurred the distinction between the former terms.

The following abbreviations are used in the manuscript:

**Male terminalia**: Aedeagus: **AEA**: ejaculatory apodeme; **AES**: aedeagal sclerite; **ALP**: lateral ejaculatory process; **AS**: sperm sac; **ASA**: subaedeagal sclerite; **AVA**: ventral arm of subaedeagal sclerite.

Epandrium: **CA**: epandrial apodeme; **ECL**: epandrial collar; **ECP**: epandrial clasper; **EL**: epandrial lobe; **EPI**: epiproct; **EPM**: subepandrial membrane; **EVL**: ventromesal lobe of epandrium; **HYP**: hypoproct.

Gonopod: **GBL**: basal lobe of gonostylus, divisions B_1_, B_2_, B_3_; **GCA**: gonocoxal apodeme; **GCT**: gonocoxite; **GDS**: dorsal spur of gonocoxal apodeme; **GLD**: dorsal gonocoxal lobe; **GPB**: primary branch of the apical lobe, divisions P_1_, P_2_; **GSB**: secondary branch of the apical lobe.

Hypandrium: **HBD**: basal division of hypandrium; **HBS**: basal scale; **HC**: hypandrial conjunctive; **HLT**: lateral lobule of terminal division; **HSL**: spathate lobe; **HTD**: terminal division of hypandrium; **HTE**: lateral extension of terminal division.

Paramere: **PMB**: paramere base; **PPA**: apical process of paramere.

**Female terminalia: CRC**: cercus; **EPG**: epigynium; **GFK**: genital fork; **HPG**: hypogynium; **HVL**: hypogynial valve; **SPT**: spermatecha.

### Barcoding

Non-destructive DNA barcoding for COI (barcoding region subunit 1) was done as outlined in Yeo et al. (2021). Briefly, DNA extraction was conducted for individual specimens with HotSHOT buffers (Truett et al. 2000), amplified via PCR with tagged primers, cleaned, and sequenced with a MinION™ (Oxford Nanopore Technologies, Oxford, UK) to assemble 313 bp barcodes using ONTBarcoder (v. 2.3.0) (Srivathsan et al. 2023). Of all the material, only two specimens yielded viable sequences, which were then uploaded to GenBank.

Specimens from the following depositories were examined:

**CKLP** Private collection of L.-P. Kolcsár

**QSBG** Queen Sirikit Botanic Garden, Chiang Mai, Thailand

**ZISP** Dipterological Collection, Zoological Institute, Russian Academy of Sciences, Saint-Petersburg, Russia

**ZRC** Lee Kong Chian Natural History Museum (Zoological Record Collection), Singapore

**USNM** Smithsonian National Museum of Natural History, Washington DC, USA

## Results

### Taxonomic treatment

***Ptychoptera formosensis* species group**

**Diagnosis**. Male genitalia with epandrial claspers elongate, apex digitiform, without ventromesal lobe. Gonostylus basal lobe divided, approximately crescent shaped; primary branch and secondary branch subequal in length, apex of gonostylus appearing bifid.

**Description**. Epandrial lobes and claspers digitiform, without point of articulation, ventromesal lobe absent. Hypoproct ovoid, subepandrial sclerite strongly developed, membrane dorsoanterior to parameres heavily sclerotized. Paramere fused to bridge, subtrapezoidal. Gonocoxite without gonocoxal lobes. Gonostylus basal lobe divided (B_1_, B_2_), crescent shaped; apical lobe divided, primary branch digitiform; secondary branch with flaglike base and stylate or triangular apex subequal in length to apical lobe, making apex of gonostylus appear bifid, medial surface of secondary branch with numerous black conical sensilla. Hypandrium with basal division bandlike, spathate lobes present, basal scale present, hypandrial conjunctive present and fused to basal division. Aedeagus tilted 45° from vertical, ejaculatory apodeme broad and fanlike, extending into preceding abdominal segment, sperm sac subspherical, lateral ejaculatory process without bend. Aedeagal sclerite rounded, constricted at base. Subaedeagal sclerite turnip-shaped to conical, posteroventral surface frequently with serrations subapically.

**Included species**. *Ptychoptera annandalei* Brunetti, 1918; *Ptychoptera emeica* Kang, Xue & Zhang, 2019; *Ptychoptera formosensis* Alexander, 1924; *Ptychoptera lii* Kang, Yao & Yang, 2013; *Ptychoptera lushuiensis* Kang, Yao & Yang, 2013; *Ptychoptera persimilis* Alexander, 1947; *Ptychoptera pseudosimilis* Fasbender, sp. nov.; *Ptychoptera tianmushana* Shao & Kang, 2021; *Ptychoptera vietnamensis* Paramonov, sp. nov.; *Ptychoptera yunnanica* Zhang & Kang, 2021.

#### Ptychoptera annandalei

Taxon classificationAnimaliaDipteraPtychopteridae

Brunetti, 1918

149B9E22-A928-5AF7-AE55-0738ACA8AE8D

Figs 2A, 3A, 4–9

Ptychoptera cordata Zhang & Kang, 2021, syn. nov.

##### Material examined.

**Thailand** • 1 male; Tak, Umphang, Umphang Wildlife Sanctuary, Mae klong kee ranger unit; 1234 m a.s.l.; 16.225548, 98.980368; 2–28.IV.2020; Malaise trap 2; J. Chomrit leg., QSBG. • 1 male; Tak, Umphang, Umphang Wildlife Sanctuary, Mae klong kee ranger unit; 1234 m a.s.l.; 16.225538°N, 98.980368°E; 30.X–27.XI.2020; Malaise trap 2; W. Srisuka leg., QSBG. • 1 female; Tak, Umphang, Umphang Wildlife Sanctuary, Mae klong kee ranger unit; 1234 m a.s.l.; 16.225538°N, 98.980368°E; 22.VII–26.VIII.2020; Malaise trap 2; W. Srisuka, M. Tabutr leg., QSBG. • 1 male; Tak, Umphang, Umphang Wildlife Sanctuary, Ancient forest; 1249 m a.s.l.; 16.245°N, 98.998348°E; 28.IV–25.VI.2020; Malaise trap 2; W. Srisuka, M. Tabutr leg., QSBG. • 1 male; Tak, Umphang, Umphang Wildlife Sanctuary, Ancient forest; 1249 m a.s.l.; 16.245°N, 98.998348°E; 22.VII–26.VIII.2020; Malaise trap 2; W. Srisuka, M. Tabutr leg., QSBG. • 3 males; Tak, Umphang, Umphang Wildlife Sanctuary, Mae klong kee ranger unit; 1231 m a.s.l.; 16.225235°N, 98.979734°E; 28.IV–25.VI.2020; Malaise trap 1; W. Srisuka, M. Tabutr leg., QSBG. • 1 male; Tak, Umphang, Umphang Wildlife Sanctuary, Mae klong kee ranger unit; 1231 m a.s.l.; 16.225235°N, 98.979734°E; 25.VI–22.VII.2020; Malaise trap 1; W. Srisuka, M. Tabutr leg., QSBG. • 1 male; Tak, Umphang, Umphang Wildlife Sanctuary, Mae klong kee ranger unit; 1231 m a.s.l.; 16.225222°N, 98.97975°E; 2–28.IV.2020; Malaise trap 2; J. Chomrit leg., QSBG. • 1 male; Chiang Rai, Huai Khun Phra, coffee plantation, muddy spring; 1190 m a.s.l.; 19.09705°N, 99.36804°E; 23.V.2024; L.-P. Kolcsár leg.; CKLP. • 1 male; Uthai Thani, I Mat stream at The Giant Argus Pheasant Tree; 640 m a.s.l.; 15.130391°N, 99.365334°E; 16.V.2024; L.-P. Kolcsár leg.; CKLP. • 3 males; Chiang Mai, Chang Khian Mai, Khun Chang Khian Highland Agricultural Research and Training Station; 1420 m a.s.l.; 18.839152°N, 98.898284°E; 25.X.2025; Kolcsár L.-P., P. Suttiprapan, P. Muenkaew leg.; CKLP. **Myanmar (Burma)** • 2 males; So. Shan States, 40 km E of Taunggyi, IX-25.IX–13.X.1934; Rene Malaise leg.; USNM.

##### Diagnosis.

Male genitalia with epandrial clasper evenly curved ventrally, apex rounded. Paramere dorsoventrally flattened, apical process semicircular with spine directed anteromedially. Apex of primary branch directed dorsomedially, base of secondary branch flag-like and subquadrate, apex triangular; spathate lobes triangular.

##### Redescription.

Coloration varies among specimens, especially on the lateral thoracic sclerites; see also the description of *Ptychoptera cordata* syn. nov. (Zhang and Kang 2021). The described color pattern represents the most commonly observed form, based mainly on specimens preserved in alcohol.

**Male. *Measurements***: Body length 7–7.4 mm, wing length 6.5–6.7 mm, width 1.6–1.7 mm (*n* = 3). Antenna 4.5–4.6 mm (*n* = 3).

**Figure 2.**
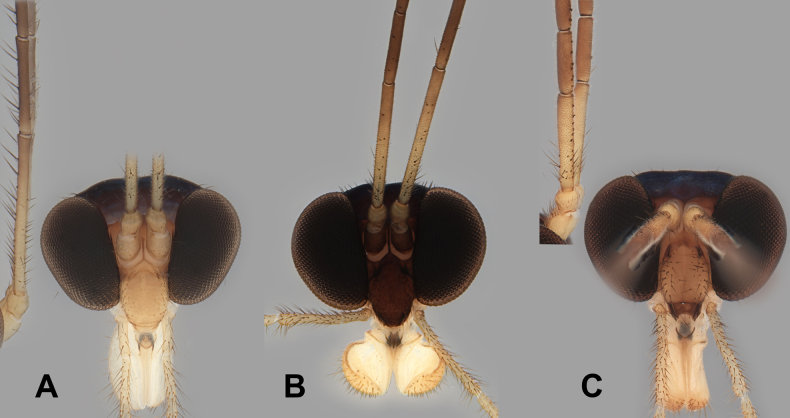
Heads. **A**. *Ptychoptera annandalei* Brunetti, 1918; **B**. *Ptychoptera perbona* Alexander, 1946; **C**. *Ptychoptera phutphi* Kolcsár & Fasbender, sp. nov.

***Head***. Vertex shining black, blue from certain angles, glabrous; frons narrow strip yellow; occiput dark brown, behind eyes yellow, glabrous, stemmatic bulla with black spot; gena narrow yellow; occiput and gena with sparse elongate brown setae; facial sclerite weakly delineated from clypeus, yellow; clypeus rhomboidal, inflated, yellow, covered with pale brown setae; palpus pale brown, last segment basally pale brown, tip dark brown; labrum spade shaped; labium yellow; labellum yellow with brown setae (Fig. 2A). Scape pale brown; pedicel pale brown; first flagellomere pale brown or apical 1/4 brown; remaining flagellum dark brown. First flagellomere 1.4–1.7× longer than second flagellomere (Fig. 2A).

**Figure 3.**
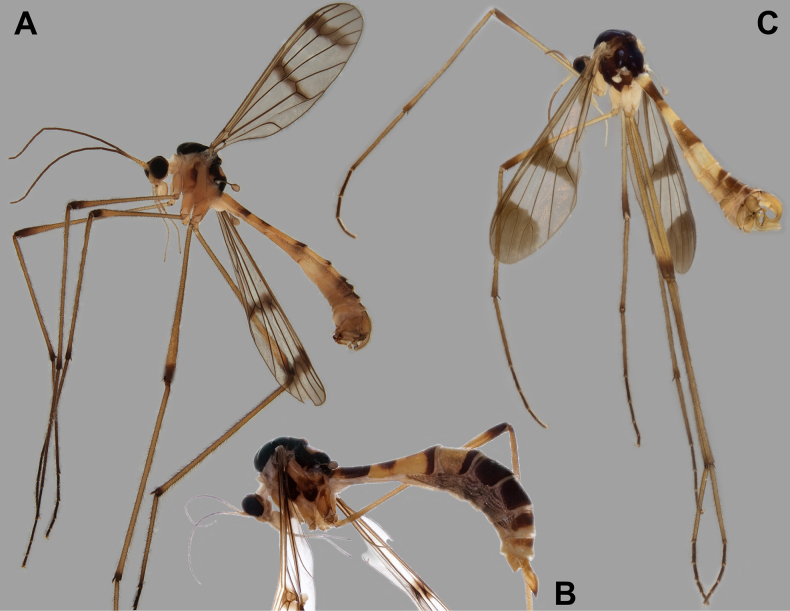
**A, B**. *Ptychoptera annandalei* Brunetti, 1918: **A**. Male, **B**. Female; **C**. *Ptychoptera phutphi* Kolcsár & Fasbender, sp. nov. male.

**Figure 4.**
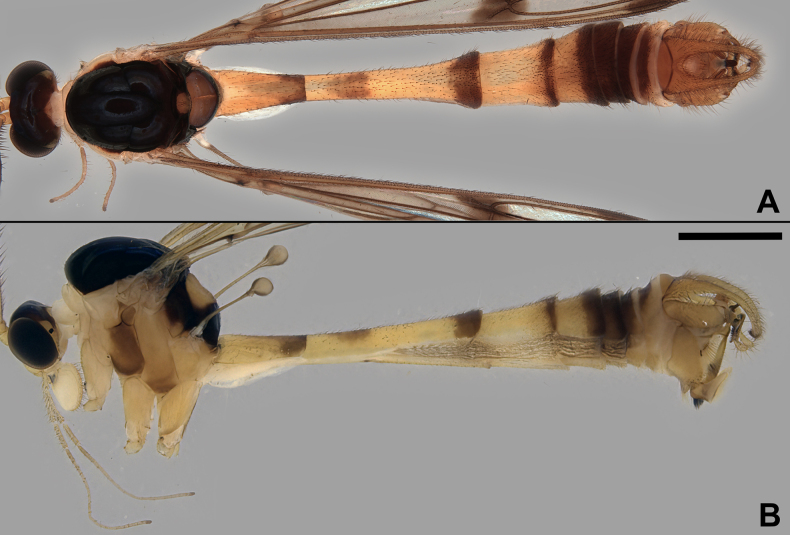
*Ptychoptera annandalei* Brunetti, 1918. Male habitus. **A**. Dorsal; **B**. Lateral. Scale bar: 1 mm.

**Figure 5.**
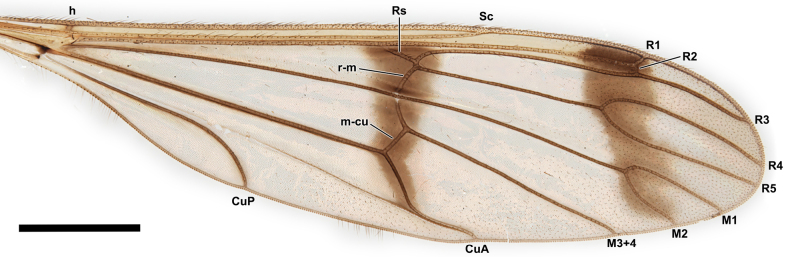
*Ptychoptera annandalei* Brunetti, 1918. Wing. Scale bar: 0.5 mm.

***Thorax***. Antepronotum yellow; postpronotum yellow (Figs 3A, 4B); prescutum uniformly shiny black, blue in certain angles; scutum uniformly shiny black, blue in certain angles; scutellum dark brown, with round pale brown spot in middle with dark brown setae; postalar callus dark brown; postalar wall dark brown; mediotergite anterior 1/2 pale brown appearing as big yellow spot in dorsal view, posterior 1/2 dark brown to black blue in certain angles (Fig. 4A); paratergite pale brown; propleuron yellow to pale brown; anepisternal cleft yellow; anepisternum yellow to brown, degree of coloration variable; katepisternum yellow to brown, degree of coloration variable; anterior basalare yellow; posterior basalare yellow to brown, degree of coloration variable; epimeron yellow to brown, ventrally always darker, degree of coloration variable; laterotergite shiny black, ventral corner pale brown, with patch of long setae; metanepisternum yellow; metakatepisternum yellow; metanotum yellow (Fig. 4). ***Wing***. Wing length/width ratio 3.9–4.1. Two brown pigment bands present; basal band absent; middle band from fork of Rs to proximal angle of CuA; subapical band not reaching wing apex, extending from pterostigma to M2; cells c pale brown; pterostigma brown (Fig. 5). Membrane with microtrichia in every cell missing or less abundant in br, bm, cua, cup up to pseudovein. Sc terminates 1.5× the length of r-m beyond fork of Rs; apex of R1 beyond fork of R_4+5_, Rs stem subequal to r-m; r-m joins R_4+5_ distal to R3 fork; R4 subequal to R_4+5_; proximal curve of R4 even arc. M_1+2_ fork 2/3 length of R_4+5_ fork. CuA sigmoidal distal to m-cu (Fig. 5). ***Halter***. Stem basal 1/3 white or yellow, apical 2/3 pale brown; knob brown (Fig. 4). Prehalter yellow (Fig. 4B). ***Leg*s**. Fore coxa yellow; fore trochanter pale brown; fore femur pale brown, apical 1/5 dark brown; fore tibia pale brown, apical 1/7 dark brown; fore tarsomere 1 brown; fore tarsomeres 2–5 dark brown. Mid coxa yellow; mid trochanter pale brown; mid femur pale brown, apical 1/6 dark brown; mid tibia pale brown, apical 1/6 dark brown; mid tarsomere 1 brown; mid tarsomeres 2–5 dark brown. Hind coxa yellow; hind trochanter pale brown; hind femur pale brown, apical 1/6 dark brown; hind tibia pale brown, apical 1/7 dark brown; hind tarsomere 1 pale brown, apical 1/5–1/6 dark brown; hind tarsomeres 2–5 dark brown (Fig. 3A).

***Abdomen***. Tergite 1 anterior 2/3 yellow, posterior 1/3 dark brown; tergite 2 yellow with a basal pale brown patch, posterior 1/5 dark brown; tergite 3 yellow or posterior margin dark brown; tergite 4 anterior 2/3 yellow, posterior 1/3 dark brown; tergite 5 brown; tergite 6 brown; tergite 7 brown. Sternite 1 yellow; sternite 2 yellow; sternite 3 yellow, without accessory copulatory organ; sternite 4 yellow; sternite 5 yellow; sternite 6 yellow; sternite 7 yellow (Fig. 4).

**Figure 6.**
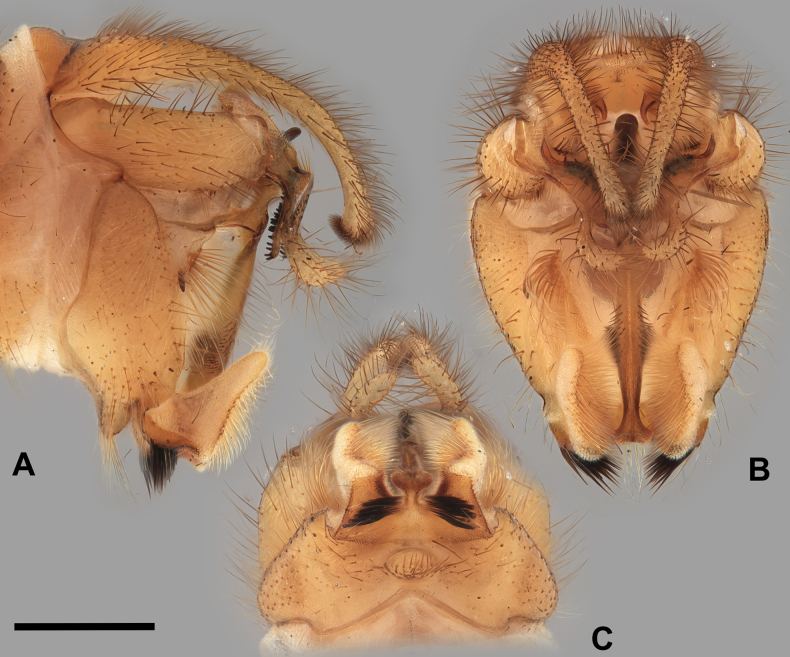
*Ptychoptera annandalei* Brunetti, 1918. Unmacerated male terminalia. **A**. Lateral; **B**. Posterior; **C**. Ventral. Scale bar: 0.5 mm.

**Figure 7.**
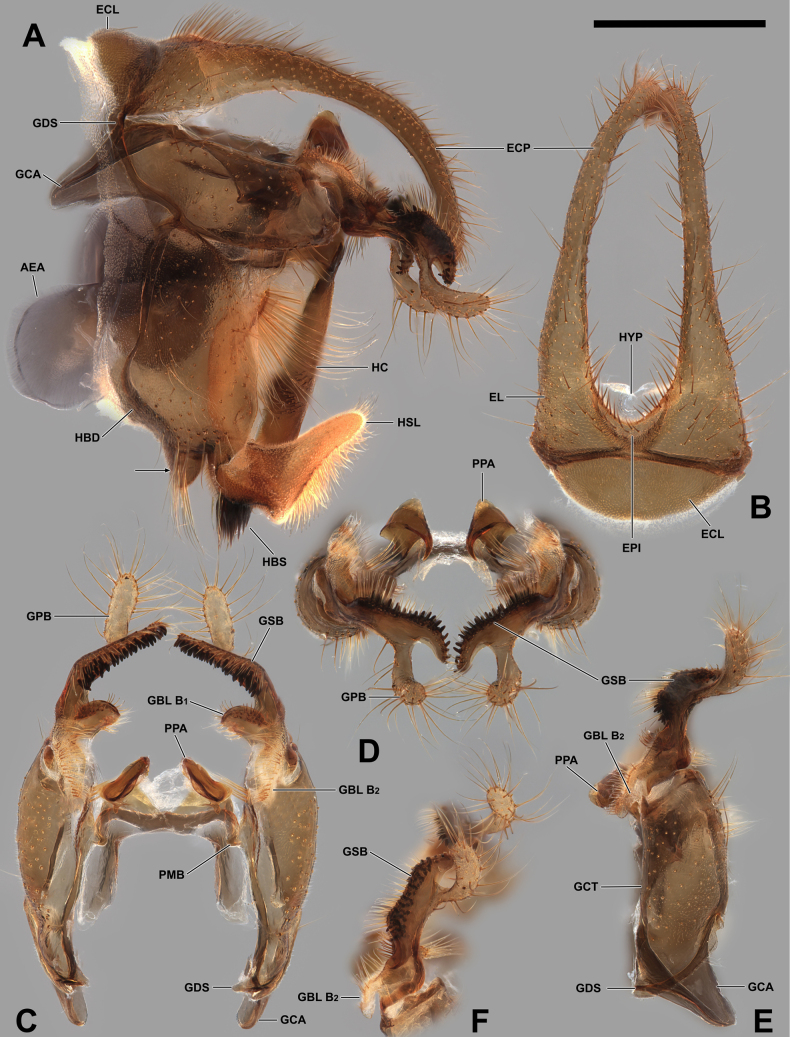
*Ptychoptera annandalei* Brunetti, 1918. Male terminalia. **A**. Overall, lateral; **B**. Epandrium, dorsal; **C**. Gonopods and parameres, dorsal; **D**. Gonopods and parameres, posterior; **E**. Gonopod, outer lateral; **F**. Gonostylus, outer later, slightly bent ventrally. Scale bar: 0.5 mm.

**Figure 8.**
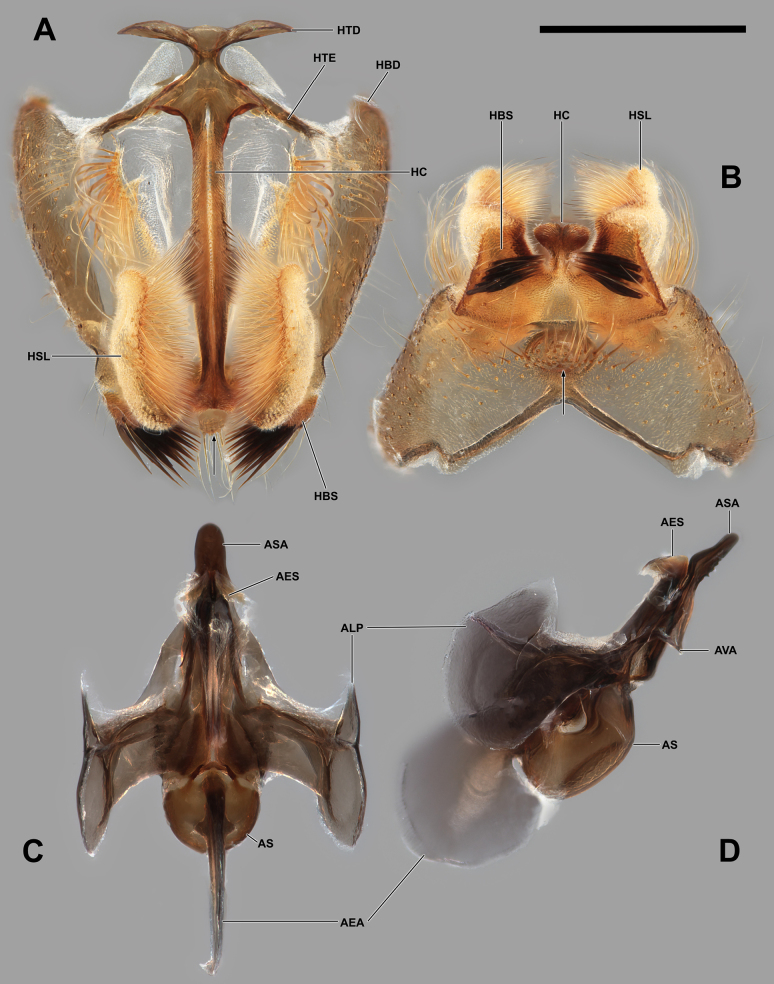
*Ptychoptera annandalei* Brunetti, 1918. Male terminalia. **A**. Hypandrium, posterior; **B**. Hypandrium, ventral; **C**. Aedeagus, anterior; **D**. Aedeagus, lateral. Scale bar: 0.5 mm.

***Male terminalia***. Pale brown (Figs 4, 6). Epandrial collar complete, well developed (Fig. 7A, B). Epandrial apodemes well developed, thread-like, extending across gonocoxal apodeme to hypandrium, with breaks present at epandrium and hypandrium (Fig. 7A). Epandrial lobes digitiform, elongate (Fig. 7B). Contiguous epandrial clasper indistinguishable from epandrial lobe, narrow, gently curved ventrally (Fig. 7A); apex rounded, slightly bulbous, bearing a ventral patch of dense short black setae (Fig. 6A). Epiproct readily apparent, posterior margin of U-shaped; in some specimens completely separating the epandrial lobes, in other the lobes are in contact on anteromedial margin. Hypoproct small sclerite, basally mostly membranous. Subepandrial membrane “n”-shaped (Fig. 7C). Parameral bridge undivided, directed posteriorly at base, then turning inward at a nearly perpendicular angle, with medial section nearly straight (Fig. 7C). A small pointed lobe present at lateral corner, directed anteriorly (Fig. 7C); apical process with narrow base and a secondary lateral spine; distally expanded into a flattened plate with distinct claw-like point, directed anterolaterally in dorsal view and ventrally in lateral view (Fig. 7C, D); in certain orientations apical margin appears semilunar (see Zhang and Kang 2021: fig. 2C). Gonocoxal apodeme triangular, subacute (Fig. 7E). Dorsal spur of gonocoxite present, nearly equilateral (Fig. 7E). Gonocoxal lobes absent; a patch of long setae present on dorsomedial margin between base of gonostylus and base of parameres; a few short black setae present on ventromedial margin in same region. Gonostylus with basal lobe divided into two branches: posterior branch (B_1_) directed 45° inward, flattened, appearing rounded (Fig. 7C) to finger-like from different angles in dorsal view, covered with short darker and longer paler setae; anterior branch (B_2_) on dorsal side elongate, laterally flattened, less chitinized, directed anteriorly in dorsal view (Fig. 7C) and anterodorsally in lateral view (Fig. 7F, E), covered with dense setae. Apical lobe divided; primary branch undivided, finger-like, expanding toward apex, directed posteriorly in dorsal view (Fig. 7C); in lateral view overlapping with secondary lobe in certain angles (Fig. 7F); secondary branch present, strongly sclerotized, flag-like and subquadrate, directed inward in dorsal view (Fig. 7C), apex triangular, weakly arched ventrally in lateral view (Fig. 7A), surface covered with stout black setae. Hypandrium with basal division rhomboid in lateral view (Fig. 7A); ventral margin with a prominent cone-shaped outgrowth (Figs 7A, 8A, 8B, arrow). Basal scale present, fully divided, fused dorsally with spathate lobe, bearing a patch of prominent, dense black setae directed ventromedially (Figs 7A, 8A, 8B). Spathate lobe large, approx. triangular, base wider, apex rounded, directed dorsally in lateral view (Figs 6A, 7A), covered with long setae directed toward other lobe; setae not overlapping, covering only ventral half of hypandrial conjunctive (Fig. 8A, B). Hypandrial conjunctive present, reaching base of terminal division, slightly dilated at mid-length, bearing black setae directed laterally toward basal scales (Fig. 8A, B). Terminal division with well-developed lateral extensions connected to basal division; apex triangular, ending in laterally directed lobes (Fig. 8A). Aedeagus tilted ~ 45° from the vertical in lateral view (Figs 7A, 8D). Aedeagal sclerite weakly tapering and rounded apically in dorsal view, subequal in length to the sperm sac (Fig. 8C); apical apodemes present, triangular in both lateral and dorsal views (Fig. 8C, D). Lateral ejaculatory processes with discoid, reniform apodemes terminating dorsally in a triangular point in lateral view (Fig. 8C), subequal in length to the ejaculatory apodeme. Sperm sac subspherical, subequal in size to the ejaculatory apodeme in lateral view (Fig. 8C). Ejaculatory apodeme subcircular, not adjacent to the base of the aedeagus (Fig. 8C), extending anteriorly into segment VII (Fig. 7A). Subaedeagal sclerite subconical, tapering posteriorly toward the Aedeagal sclerite, ventrally with sawtooth-like serrations (Fig. 8D), ventral arm of subaedeagal sclerite present, triangular.

**Female. *Measurements***: Body length 7.5 mm, wing length 6.7 mm, width 1.6 mm (*n* = 1) Antenna 4 mm (*n* = 1).

***Head***. As in male. First flagellomere 1.3× longer than second flagellomere.

***Thorax***. In general, as in male. Thorax of studied specimen darker than those in males. ***Wing***. As in male. Wing length/width ratio 4.1. ***Legs***. As in male.

**Figure 9.**
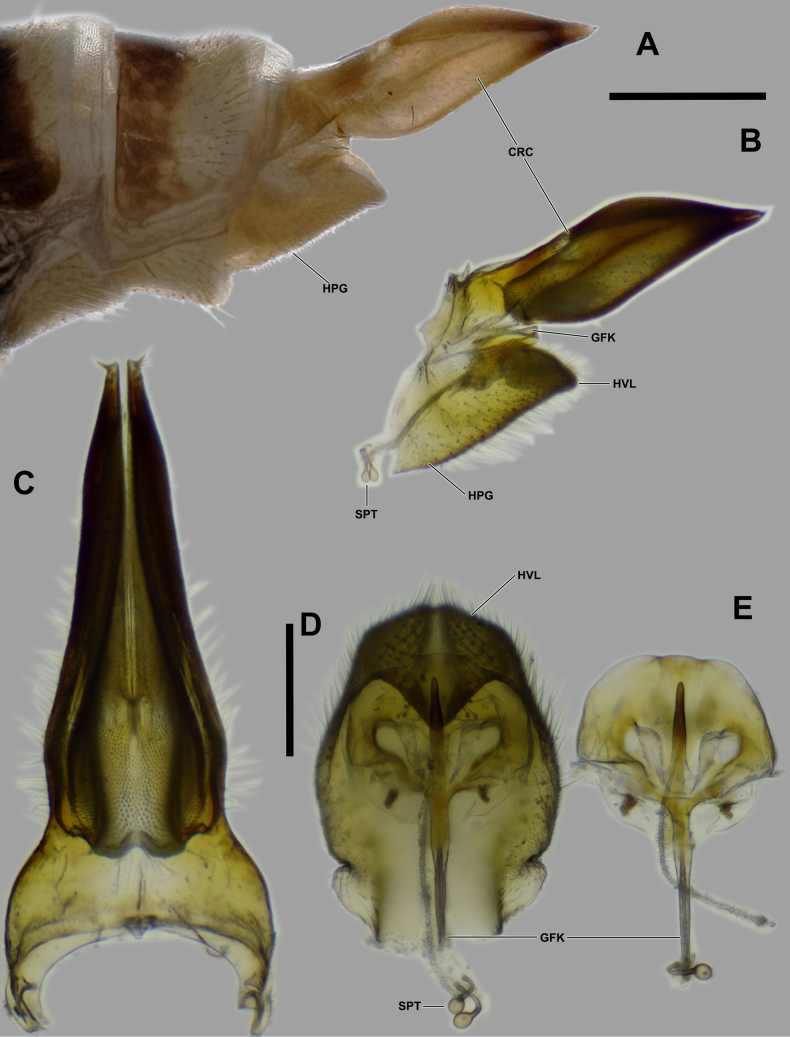
*Ptychoptera annandalei* Brunetti, 1918. Female terminalia. **A**. Unmacerated, lateral; **B**. Macerated, lateral; **C**. Epigynium and cerci, ventral; **D**. Hypogynium, hypogynial valves and genital plate, dorsal; **E**. Genital plate. Scale bars: 0.5 mm (**A, B**); 0.25 mm (**C–E**).

***Abdomen***. Tergite 1 anterior 1/3 pale brown, posterior 2/3 black; tergite 2 yellow with a basal dark brown patch, posterior 1/5 dark brown; tergite 3 yellow, posterior 1/5 dark brown; tergite 4 dark brown; tergite 5 dark brown; tergite 6 anterior 1/2 brown, posterior 1/2 whitish yellow; tergite 6 anterior 1/2 pale brown, posterior 1/2 whitish. Sternite 1 yellow; sternite 2 yellow; sternite 3 yellow, without accessory copulatory organ; sternite 4 yellow; sternite 5 yellow; sternite 6 yellow; sternite 7 yellow (Fig. 9C).

***Female terminalia***. Hypogynium without a lobe at the corner (Fig. 9A, B). Separation between the hypogynium and hypogynial valves indistinct; apex with a shallow incision (Fig. 9D). Cerci grain-shaped. Sternite 9 with the posterior margin forming a rounded plate bearing a small, weakly distinct median rounded lobe (Fig. 9E). Bursa dark, needle-shaped, with two ear-shaped lateral invaginations and darkened anterior ends (Fig. 9D, E). Two (possibly three) spermathecae, very small and spherical, approx. as wide as the genital fork in lateral view (Fig. 9B, D, E).

##### Distribution.

China (Yunnan), Myanmar/Burma (Shan States), Thailand (northern Thailand).

##### Remarks.

The species was originally described from Myanmar (Burma) from Shan State by Brunetti (1918). The material determined by Alexander and figured in Fasbender (2014) was crushed in the process of slide mounting, which distorted the features of the gonostylus and epandrium. The new material we report here allowed us to examine these features in their undistorted state, and examination of the genitalia from different angles revealed that *Ptychoptera cordata* Zhang & Kang, 2021 is a junior synonym of this species, and that the previously reported differentiating characters—such as the overlapping primary branch and secondary branch of the gonostylus and the shape of the parameres—are the result of viewing these structures from different angles. It is now clear that *P. annandalei* is a member of the *P. formosensis* group based on the structure of the gonostylus.

#### Ptychoptera persimilis

Taxon classificationAnimaliaDipteraPtychopteridae

Alexander, 1947

471A63E5-D77A-5170-A05D-780BB0AF91F5

Figs 10, 11

##### Type material.

***Holotype*. Myanmar** • male; Southern Shan State, Shwenyaung; [no coordinates]; viii.1930; [no leg.], USNM.

##### Diagnosis.

Male genitalia with epandrial claspers having posterior curve in apical portion, apex slipper-shaped. Paramere dorsoventrally flattened with elongate ventral spines that cross medially. Gonostylus with apex of primary branch nearly straight and with slightly bulbous apex, base of secondary branch flag-like and rounded, apex thumb-shaped.

##### Redescription.

**Male. *Measurements***: Body length 8 mm; wing length 7 mm (from Alexander 1947).

***Head***. Unknown.

**Figure 10.**
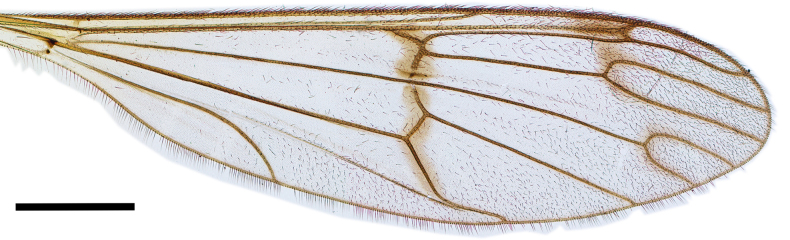
*Ptychoptera persimilis* Alexander, 1947. Wing. Scale bar: 0.5 mm.

***Thorax***. Scutum black, scutellum yellow, adjoining portion of mediotergite of metanotum yellow; pleural sclerites yellow, mesepisternum darker (from Alexander 1947). ***Wing***. Wing length/width ratio 3.5. Two pigment bands present, basal band absent; middle band from Rs to medial curve of CuA, apical band from R_1+2_ fork to R_4+5_ fork, with spot at M fork. Membrane with macrotrichia in all cells except for proximal portions of br, bm, cua and cup. All veins covered in short stout macrotrichia. Sc terminates 1.5× the length of r-m, beyond fork of Rs. Rs shorter than r-m; r-m joins R_4+5_ distal to R_3_ fork; R_4+5_ stem and R_4_ subequal, proximal curve of R_4_ squared off. M_1+2_ fork ~ 1/2 as long as R_4+5_ fork. CuA sigmoidal distal to m-cu (Fig. 10). ***Halter***. Unknown. ***Legs***. Femora yellow with distal apex infuscated, tibia yellow with distal apex darkened, tarsomere 1 yellow with distal apex black, remaining tarsomeres black (from Alexander 1947).

***Abdomen***. Tergite 1 black, tergite 2 yellow with proximal and distal margins dark, remaining tergites yellow with dark posterior band. Sternites and terminalia yellow (from Alexander 1947).

**Figure 11.**
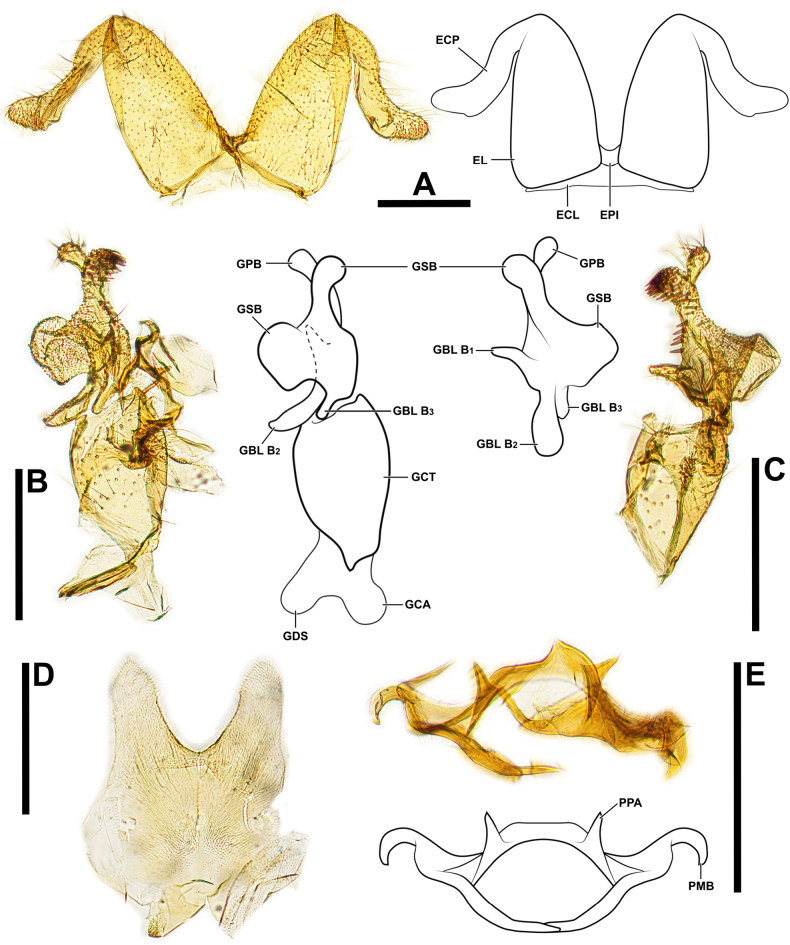
*Ptychoptera persimilis* Alexander, 1947. **A**. Epandrium, dorsal; **B**. Gonopod, inner lateral; **C**. Gonostylus, outer lateral; **D**. Subepandrial plate; **E**. parameres. Scale bars: 0.5 mm.

***Male terminalia***. Epandrial collar complete, closely appressed to epandrial lobes (Fig. 11A). Epandrial apodemes well developed, thread-like across gonocoxal apodeme to hypandrium, breaks present at epandrium and hypandrium. Epandrial lobes digitiform, basal half broad, very weakly tapering, longer than width of epandrium; epandrial clasper not well delineated from epandrial lobe, apex of epandrial clasper curved posteriorly, slipper-shaped (Fig. 11A). Epiproct very reduced, posterior margin simple shallow U-shaped notch, completely separating epandrial lobes (Fig. 11A). Posteromedial stylus absent. Hypoproct indistinct. Subepandrial membrane weakly sclerotized, weakly attached to epandrium, quadrate, paired posterior lobes present, lobe 1.5× longer than wide at base, gently narrowing toward the tip, margins almost parallel, tip triangular, emargination V-shaped (Fig. 11D). Parameral bridge attached narrowly to gonocoxite, arced weakly dorsally; ventroposterior margin with medially directed elongate flattened spine placed near base (Fig. 11E). Apical process placed dorsally, short triangular spine 2/3 distance between base and middle of paramere (Fig. 11E). Gonocoxal apodeme weakly tapering, apex rounded, greater than half length of gonocoxite. Dorsal spur of gonocoxal apodeme present, subequilateral triangular, shifted posterior onto anterodorsal margin of gonocoxite, nearly as long as remainder of gonocoxal apodeme (Fig. 11B). Gonocoxite ovoid, dorsal margin heavily arced dorsally, ventral margin shallowly convex (Fig. 11B). Gonocoxal lobes absent, a patch of long setae present on dorsomedial margin between base of gonostylus and base of parameres; a few short black setae present on ventromedial margin in same region. Gonostylus with basal lobe divided into three branches: posterior branch (B_1_) approx. finger-shaped, dorsal branch (B_2_) laterally flattened tongue-to finger-like from different angles; B_1_ and B_2_ form crescent shape; B_3_ finger-like at base of B_2_ (Fig. 11B, C). Primary branch finger-like, round in cross section, apex curved laterally, weakly expanding to a setose knob at apex (Fig. 11B, C). Secondary branch subdivided, basal portion sail-like, round, laterally compressed, directed dorsally, covered in fine trichoid sensilla; posterior portion finger-like, directed posterior, curving medially, expanding at apex, with numerous stout, blackened trichoid sensilla on medial surface apically (Fig. 11B, C). Distal portion of secondary branch and primary branch with apices crossing (Fig. 11B, C). Hypandrium damaged on specimen examined. Spathate lobes present, originating near midline, broadly elongate with rounded apex. Basal scale presence uncertain. Terminal division with pentagonal apex, lateral apices quadrate, with ventrally curved spine. Hypandrial conjunctive present, shape uncertain. Aedeagus orientation not readily apparent in holotype. Aedeagal sclerite with apex pointed, simple. Subaedeagal sclerite with constriction at apex of Aedeagal sclerite, apex turnip shaped, lateral margins seem straight, tapering. Sperm sac damaged. Ejaculatory apodeme and lateral ejaculatory processes not readily diagnosed in specimen examined.

##### Distribution.

Myanmar (Shan State).

##### Remarks.

*Ptychoptera persimilis* is a member of the *P. formosensis* group. It is very similar to *P. lushuiensis*, both species having a similar wing banding pattern and the apex of the epandrial claspers curved posteriorly. We have differentiated them primarily based on details of the genitalia: the primary branch of the gonostylus is weakly curved laterally with the tip expanding slightly in *P. persimilis*, while the illustration of *P. lushuiensis* (Kang et al. 2013; Yang et al. 2024) shows that structure hooked medially. Additionally, *P. persimilis* has elongated spines originating from the ventral surface of the parameres that cross medially, which are absent in the illustrations of *P. lushuiensis*. However, both of these characters may be artifacts of the compressed slide mount of the *P. persimilis* holotype, and additional examination of *P. lushuiensis* is warranted to confirm that they are distinct species. There are also strong similarities with *P. pseudosimilis*, though there are a number of characters that can be used to distinguish both taxa that are discussed in the remarks on that species.

#### Ptychoptera pseudosimilis

Taxon classificationAnimaliaDipteraPtychopteridae

Fasbender sp. nov.

91C91581-6501-5BAC-B0CC-753285532CC3

https://zoobank.org/DFB9335C-0208-4AC9-BAC6-D33FA9B5F179

Figs 12, 13

##### Type material.

***Holotype*. India** • male; Arunachal Pradesh, Kameng, Nyukmadong; 2440 m a.s.l.; [no coordinates]; 21.iv.1961; Schmid leg., USNM. HOLOTYPE *Ptychoptera pseudosimilis* Fasbender, det. A Fasbender 2014 [red label]. ***Paratype*. India** • male; Arunachal Pradesh, Kameng, Shergaon; 1950 m a.s.l.; [no coordinates]; 8.v.1961; Schmid leg., USNM.

##### Diagnosis.

Male genitalia with evenly curved epandrial claspers, apex bulbous. Paramere dorsoventrally flattened without ventral spines, triangular dorsal apices present. Gonostylus with apex of primary branch hooked with cylindrical apex, base of secondary branch flag-like and rounded, apex thumb-shaped. Hypandrium with spathate lobes tongue-like with rounded apex.

##### Description.

**Male. *Measurements***: Wing length 8.4 mm (remainder of measurements unknown due to fragmentary nature of specimens).

***Head***. Unknown.

**Figure 12.**
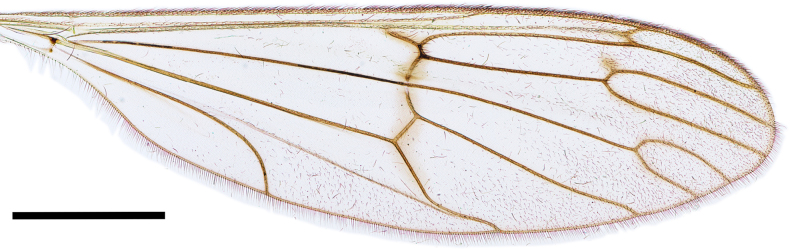
*Ptychoptera pseudosimilis* Fasbender, sp. nov. Wing. Scale bar: 0.5 mm.

***Thorax***. Unknown. ***Wing***. Wing length/width ratio 3.5. One weak pigment band present, basal and apical bands absent. Middle band from Rs to medial curve of CuA, additional small round spots at R_1+2_ fork and basal portion of R_4_. Membrane with macrotrichia in all cells except for br; br has only a line of macrotrichia medially from halfway along its length, cup and a1 only with macrotrichia distally. Sc terminates 1.5× the length of r-m, beyond fork of Rs; Rs subequal to r-m; r-m joins R_4+5_ distal to R_3_ fork; R_4+5_ stem and R_4_ subequal; proximal curve of R_4_ even arc. M_1+2_ fork 2/3 as long as R_4+5_ fork. CuA sigmoidal distal to m-cu (Fig. 12). ***Halter***. Unknown. ***Legs***. Fore and mid coxae yellow with dark band on dorsal margin, hind coxae black. Femur and tibia yellow. Tarsomere 1 yellow proximally, turning brown distally. All other tarsomeres brown. Tarsomere 5 can fold back on penultimate tarsus. Mid and hind tarsomere 1 with widely spaced row of minute, spine-like trichoid sensilla. (From slide mounted specimen).

***Abdomen***. Unknown.

**Figure 13.**
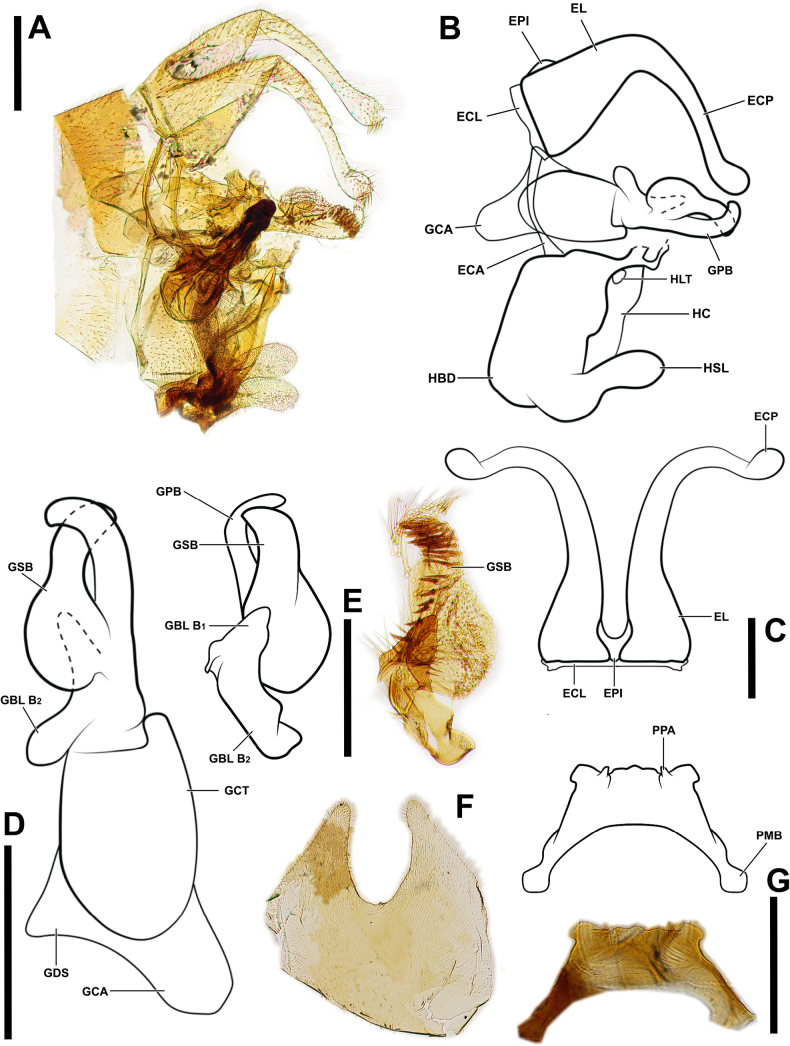
*Ptychoptera pseudosimilis* Fasbender, sp. nov. **A, B**. Overall, lateral; **C**. Epandrium, dorsal; **D**. Gonopod, outer lateral; **E**. Gonostylus, inner lateral; **F**. Subepandrial plate; **G**. Parameres. Scale bars: 0.5 mm (**A–E**); 0.25 mm (**F, G**).

***Male terminalia***. Epandrial collar complete, closely appressed to epandrial lobes (Fig. 13C); epandrial apodemes well developed, thread-like across gonocoxal apodeme to hypandrium, breaks present at epandrium and hypandrium (Fig. 13A, B). Epandrial lobes digitiform, elongate, narrow; epandrial clasper not well delineated from epandrial lobe, apex strongly expanded, rounded bulb, epandrial lobes moderately curved ventrally without posterior angle apically (Fig. 13A–C). Epiproct triangular, posterior margin simple, evenly emarginate, epandrial lobes with point contact on anterior margin (Fig. 13C). Posteromedial stylus absent. Hypoproct indistinct. Subepandrial membrane weakly sclerotized, quadrate, paired posterior lobes present, lobe as long as wide at base; inner margin gently curved inward; outer margin almost straight but directed inward; lobe gradually narrowing toward the tip; tip rounded; emargination U-shaped (Fig. 13G). Parameral bridge attached narrowly to gonocoxite, trapezoidal in dorsal view; posterior margin simple arc, anterior margin with lateral portions straight and directed posteromedially, angled medially at half width from base to midpoint, angle with flattened subquadrate corner, medial portion with low triangular lobes laterally, low semicircular lobe medially (Fig. 13G); apical process placed dorsally, short triangular spine two-thirds distance between paramere base and medial point (Fig. 13G). Gonocoxal apodeme weakly tapering, apex rounded, greater than half length of gonocoxite (Fig. 13D). Dorsal spur of gonocoxal apodeme subequilateral triangle, shifted posterior onto anterodorsal corner of gonocoxite, nearly as long as remainder of gonocoxal apodeme (Fig. 13D). Gonocoxite ovoid, dorsal margin heavily arced dorsally, ventral margin weakly convex (Fig. 13D). Gonocoxal lobes absent. Gonostylus with basal lobe divided into three branches: posterior branch (B_1_) approx. finger-like to triangular from different angles; dorsal branch (B_2_) laterally flattened, approx. tongue-like; B_1_ and B_2_ form crescent shape (Fig. 13D, E). Primary branch fingerlike, round in cross section, apex hooked laterally, cylindrical in cross-section, not expanding strongly to apex (Fig. 13D, E). Secondary branch subdivided, basal portion directed dorsally, laterally compressed, round, broader than remainder of apical lobe complex, covered with fine trichoid sensilla; posterior portion fingerlike, directed posterior, curving medially, expanding at apex, with numerous stout black conical trichoid sensilla on medial surface apically. Posterior portion of secondary branch and primary branch with apices crossing (Fig. 13D, E). Hypandrium damaged. Basal division with spathate lobes present, near medial point, tongue-like with rounded apex. Terminal division apex obscured on specimen, lateral lobule of terminal division present (Fig. 13A, B). Hypandrial conjunctive slit-like, stretching nearly entire length of terminal division (Fig. 13A). Aedeagus orientation not readily apparent in holotype. Aedeagal sclerite with apex pointed, simple. Subaedeagal sclerite with constriction at apex of aedeagal sclerite, apex turnip shaped, lateral margins rounded, tapering (Fig. 13A). Sperm sac oblong. Ejaculatory apodeme flag-like, smaller than sperm sac, ventral margin paralleling anterior margin of sperm sac. Lateral ejaculatory processes not readily diagnosed in specimen examined.

##### Distribution.

India (Arunachadal Pradesh).

##### Etymology.

The species epithet *pseudosimilis*, from the Greek *pseudo*- (false) and Latin *similis* (similar), refers to the resemblance of this species to *P. persimilis*. The epithet is a noun in apposition.

##### Remarks.

*Ptychoptera pseudosimilis* belongs to the *P. formosensis* group (see Discussion), an assemblage of species which are difficult to differentiate from each other. It is most similar to *P. persimilis*, but can be distinguished by the wing with only one pigment spot at the R_4+5_ fork (versus a broken band extending from the terminus of R_1_ to the M fork in *P. persimilis*) the apex of the epandrial claspers being bulbous (slipper-like in *P. persimilis*), the epiproct being triangular (being very reduced and V-shaped in *P. persimilis*), and the primary branch of the gonostylus being more strongly curved than in *P. persimilis*, and the apex not expanded. *Ptychoptera lushuiensis* Kang, Yao & Yang, 2013 is also similar, but differs in the same wing pattern character and also that the spathate lobes of the hypandrium are narrow and taper to a point, while those of *P. pseudosimilis* are broadly tongue-like and have a broadly rounded apex.

#### Ptychoptera vietnamensis

Taxon classificationAnimaliaDipteraPtychopteridae

Paramonov sp. nov.

0CDEF94C-7A01-58F0-9258-977D13C2377A

https://zoobank.org/C1629931-C5E4-45A1-BD58-E4E8BC9E439C

Figs 14C, 15–18

##### Type material.

***Holotype*. Vietnam** • male; Lai Châu Prov., Hoàng Liên N.P., 22.34997°N, 103.76818°E, 1947 m, 16.IV.2012, leg. D.I. Gavryushin; ZISP. HOLOTYPE *Ptychoptera vietnamensis* Paramonov, sp. nov. [red label].

##### Diagnosis.

Male terminalia with medially curved epandrial claspers with opposed apices with posterior directed swelling on the medial surface.

##### Description.

**Male. Measurements**: Wing length 8.5 mm, width 2.2 mm (*n* = 1). Antenna 4.8 mm (*n* = 1).

**Figure 14.**
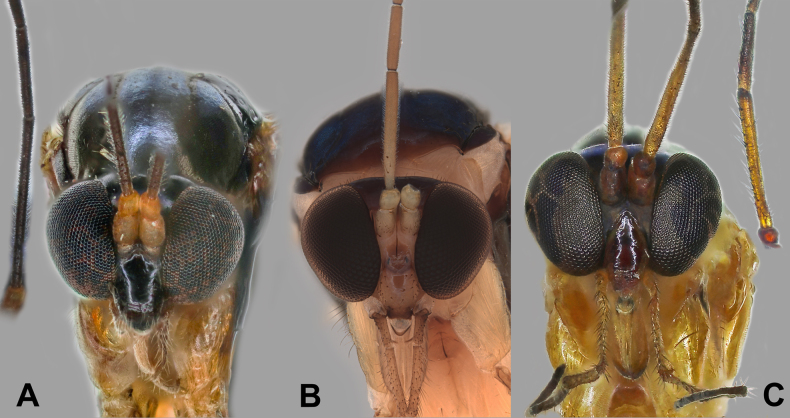
Heads. **A**. *Ptychoptera srilankaensis* Paramonov, sp. nov. **B**. *Ptychoptera hantu* Kolcsár & Fasbender, sp. nov. **C**. *Ptychoptera vietnamensis* Paramonov, sp. nov.

**Figure 15.**
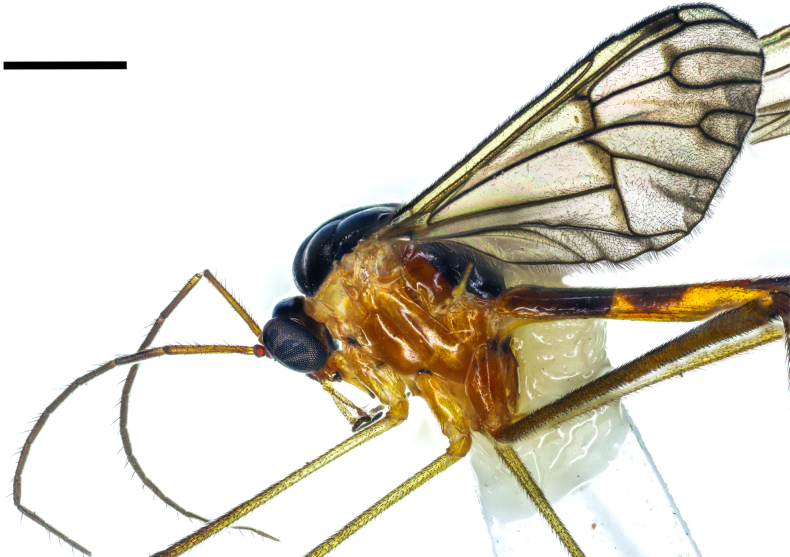
*Ptychoptera vietnamensis* Paramonov, sp. nov. Male habitus. Scale bar: 1 mm.

***Head***. Vertex shining black, blue from certain angles, glabrous; frons extremely narrow strip dark brown; occiput black, behind eyes with a brown elliptical spot (stemmatic bulla); gena very narrow black; occiput without setae, gena with brown setae; facial sclerite small, dark brown; clypeus rhomboidal, inflated, brown, with few dark brown sensilla; palpus yellow, last segment brown; labrum narrowly spade or pentagonal shaped, anterior margin dark brown; labium pale brown; labellum yellow with brown setae (Figs 14C, 15). Scape, pedicel yellow-brown; first flagellomere pale brown yellow, remaining flagellum brown, setae on antenna dark brown; first flagellomere 1.8× longer than second flagellomere (Figs 14C, 15).

**Figure 16.**
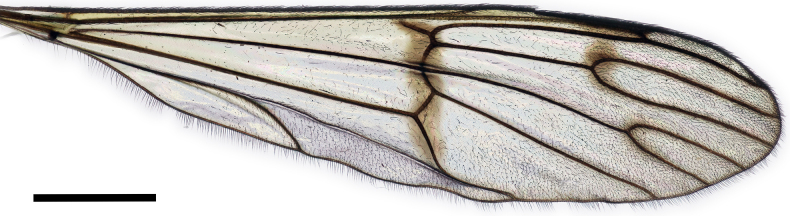
*Ptychoptera vietnamensis* Paramonov, sp. nov. Wing. Scale bar: 0.5 mm.

***Thorax***. Antepronotum yellow; postpronotum yellow; prescutum uniformly shiny black; scutum uniformly shiny black; scutellum yellow-brown, with short dark brown setae; postalar callus brown; postalar wall brown; mediotergite yellow-brown, dark brown on lateral and distal edge; paratergite yellow; propleuron yellow; anepisternal cleft yellow; anepisternum yellow; katepisternum yellow, anterior basalare yellow; posterior basalare yellow; epimeron yellow; laterotergite yellow to brown with a black stripe along the edge adjacent to the mediotergite, with patch of setae; metanepisternum yellow; metakatepisternum yellow; metanotum yellow (Fig. 15). Dark spot between antepronotum, pleuron and fore coxa; dark spot between katepisternum and mid coxa (Fig. 15). ***Wing***. Wing length/width ratio 3.8. Two pigment bands present, basal band absent; middle band from Rs to medial curve of CuA, apical band from R_1+2_ fork to R_4+5_ fork, with spot at M fork (Fig. 16). Membrane with macrotrichia in all cells except for proximal portions of br, bm, cua, and cup. All veins covered in short stout macrotrichia. Sc terminates 2× the length of r-m, beyond fork of Rs. Rs subequal or slightly longer than r-m; r-m joins R_4+5_ distal to R_3_ fork; R_4+5_ stem longer then R_4_, proximal curve of R_4_ squared off. M_1+2_ fork ~ 1/2 as long as R_4+5_ fork. CuA sigmoidal distal to m-cu (Fig. 16). ***Halter***. Stem basal 1/4 pale yellow, apical 3/4 brown (Fig. 15); knob brown. Prehalter yellow (Fig. 15). ***Legs***. Fore coxa yellow; fore trochanter yellow (Fig. 15); fore femur yellow, apical 1/2 dark brown; fore tibia brown; fore tarsomeres 1–5 brown. Mid coxa yellow; mid trochanter yellow (Fig. 15); mid femur yellow, apical 1/2 dark brown; fore tarsomeres 1–5 brown. Hind coxa yellow; hind trochanter yellow; hind femur pale brown, apical 1/8 dark brown (Fig. 15); hind tibia pale brown; hind tarsomere 1 pale brown; hind tarsomeres 2–5 dark brown.

***Abdomen***. Tergite 1 anterior 1/4 yellow, posterior 3/4 brown; tergite 2 brown, with yellow band on the middle (Fig. 15); tergites 3 and 4 yellow, distally with black spot; tergites 57 black; sternite 1 yellow; sternite 2 anterior 2/3 yellow, posterior 1/4 brown; sternite 3 yellow, without accessory copulatory organ; sternite 4 yellow; sternites 57 black.

**Figure 17.**
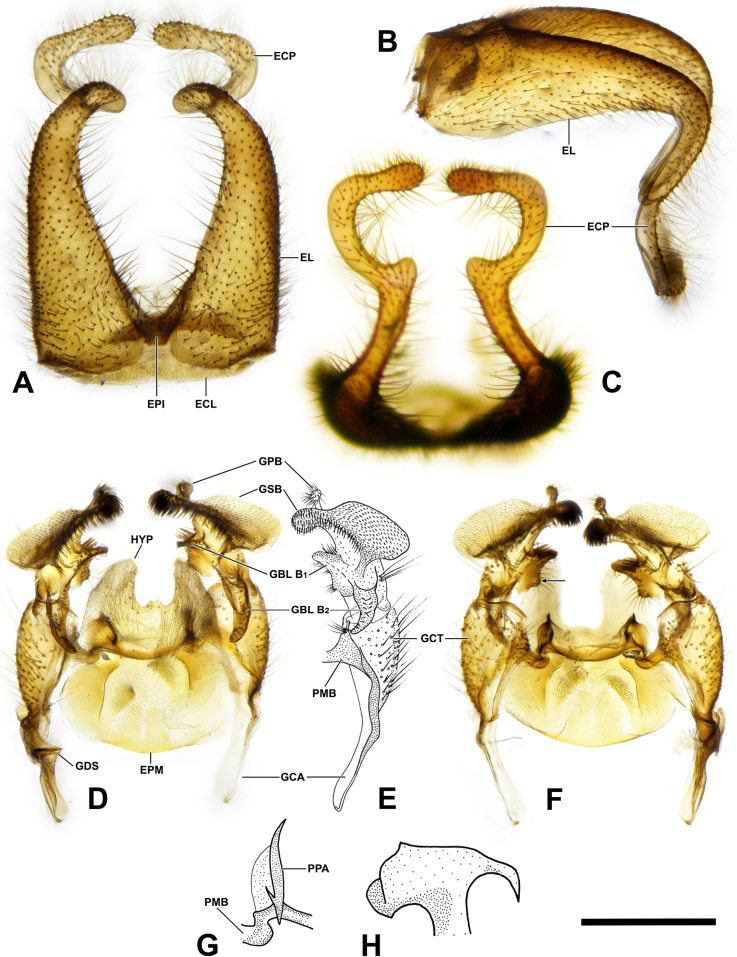
*Ptychoptera vietnamensis* Paramonov, sp. nov. **A**. Epandrium, dorsal; **B**. Epandrium, lateral; **C**. Epandrium, posterior; **D**. Gonopods and parameres, dorsal; **E**. Gonopod, dorsal; **F**. Gonopods and parameres, ventral; **G**. Paramere, dorsal; **H**. Paramere, lateral. Scale bars: 0.5 mm.

***Male terminalia***. Yellow. Epandrial apodemes well developed, thread-like across gonocoxal apodeme to hypandrium, breaks present at epandrium and hypandrium. Epandrial lobes digitiform, twice as long as width, tapering towards apex (Fig. 17A, B). Epandrial clasper contiguous with epandrial lobe, sharply down curved at ~ 90° at mid-length, slightly incurved (Fig. 17B), distal fourth of epandrial clasper sharply outcurved in semicircular shape, with medial swelling at base of curve (Fig. 17A, C); bearing prominent long setae. Apex of epandrial clasper slightly enlarged, directed medially (Fig. 17A, C). Epandrial clasper brown in basal half, yellow in distal half. Epiproct triangular, not heavily incised on posterior margin (Fig. 17A), completely separating epandrial lobes. Subepandrial membrane weakly sclerotized, weakly attached to epandrium, quadrate, paired posterior lobes present, lobe as wide as long; inner margin straight, outer margin curved medially; tip slightly squared or pointed medially; posterior margin of emargination with a triangular outgrowth, tip notched (Fig. 17D). Parameral bridge narrowly attached to gonocoxite, curved dorsoanteriorly (Fig. 17F). Apical process of paramere attached dorsally near lateral margin, tip pointed in dorsal view (Fig. 17F, G), shaped like a fire axe in lateral view: anterior part broad, posterior part narrow, tapered to hook (Fig. 17H). Gonocoxal apodeme long and narrow. Gonocoxite broadest posteriorly. Gonocoxal lobes absent; a patch of setae present on dorsomedial margin between base of gonostylus and base of parameres; a few short setae present on ventromedial margin in same region. Gonostylus with basal lobe divided into two branches: posterior branch (B_1_) pyramidal, with 6–7 ventroposteriorly directed spines anteriorly (Fig. 17D–F), with a low setose knob medially (Fig. 17F, arrow); anterior branch (B_2_) finger shaped and directed dorsoanteriorly (Fig. 17D, E).

**Figure 18.**
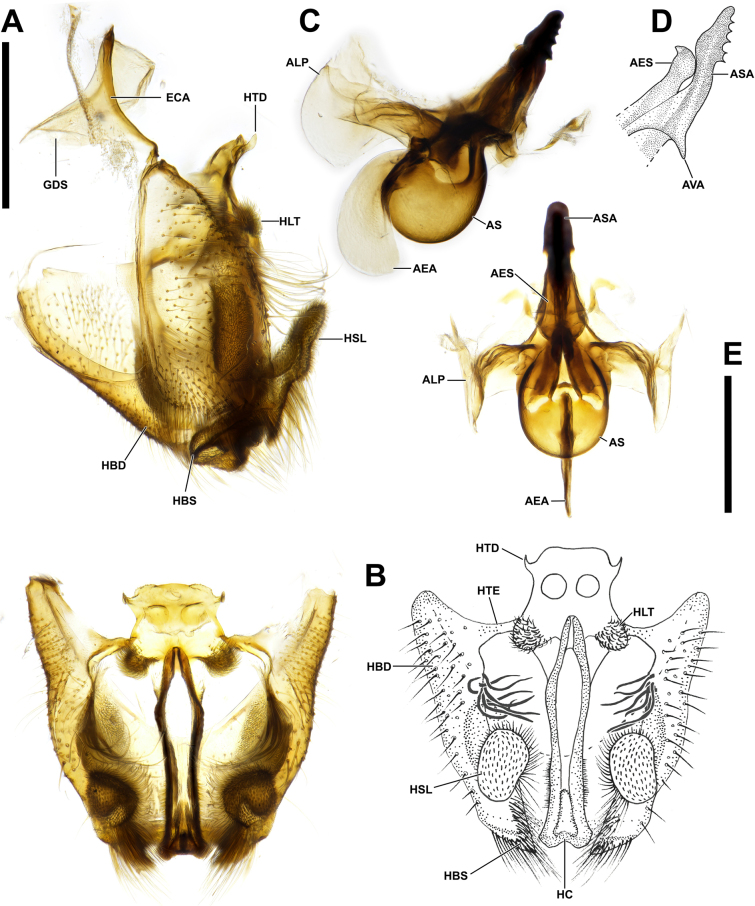
*Ptychoptera vietnamensis* Paramonov, sp. nov. **A**. Hypandrium, lateral (slightly rotated); **B**. Hypandrium, posterior; **C**. Aedeagus, lateral; **D**. Tip of aedeagus, lateral; **E**. Aedeagus, anterior. Scale bars: 0.5 mm (**A, B**); 0.5 mm (**C–E**).

Primary branch of gonostylus finger-like, round in cross section, directed posterior, curving dorsally, apex terminating in small knob (Fig. 17D–F). Secondary branch subdivided, base trapezoidal, laterally compressed, directed dorsally, with short fine trichoid sensilla, posterior portion paddle-like, laterally compressed, with numerous blackened trichoid sensilla on medial surface apically (Fig. 17D–F). Hypandrium basal division with basal scale present, fully divided, fused dorsally with spathate lobes, bearing a patch of prominent, dense black setae directed ventromedially (Fig. 18B). Spathate lobes present, blade-shaped, with apex directed dorsally and semicircular bulge midway along posterior margin; edge with long hair (Fig. 18A B). Terminal division of hypandrium apex subquadrate, lateral apices with dorsoposteriorly curved spine (Fig. 18B); lateral lobule of terminal division present (Fig. 18A, B); lateral extension of terminal division fused with basal division (Fig. 18B). Hypandrial conjunctive present, ribbonlike, stretching nearly entire length of terminal division. Aedeagal sclerite with apical apodeme; lateral ejaculatory processes with discoid apodeme scallop shaped, dorsal portion subequal to ventral portion, smaller than ejaculatory apodeme in lateral view (Fig. 18C–E). Sperm sac ovoid, subequal in length to ejaculatory apodeme in lateral view (Fig. 18C). Ejaculatory apodeme broom shaped, curved ventrally around anterior margin of sperm sac, apex broad (Fig. 18C). Subaedeagal sclerite triangular, serrate ventrally, with four points (Fig. 18C, D); ventral arm of subaedeagal sclerite present, acute spine.

**Female**. Unknown.

##### Distribution.

Vietnam (northwest).

##### Etymology.

The species epithet is given in honor of the English name of the country Vietnam; an adjective in apposition.

##### Remarks.

*Ptychoptera vietnamensis* is a member of the *P. formosensis* group based on the structure of the gonostylus. The distinctive medially curved epandrial claspers with opposed apices are found in three other species: *P. emeica*, *P. lii*, and *P. tianmushana*. It can be readily separated from these three species by the presence of a posterior directed swelling on the medial surface of the epandrial clasper where it begins to curve laterally.

### Key to the *Ptychoptera formosensis* group

**Table:** 

1	Apical lobe of gonostylus appearing forked by separation between primary branch and secondary branch which subequal in length, apex of secondary branch with numerous black conical sensilla (Figs 7C, 7D, 7F, 11B, 11C, 13D, 13E, 17D–F)	**2** (***Ptychoptera formosensis*** group)
–	Apical lobe of gonostylus not forked (if divided lobes of unequal length), apex of secondary branch with or without sensilla	**other *Ptychoptera*** (not keyed here)
2	Epandrial clasper subapically with medially directed spine or swelling subapically (Fig. 17A, C)	**3**
–	Epandrial clasper without medially directed spine	**4**
3	Posterior process of paramere triangular (Fig. 17F)	***Ptychoptera vietnamensis* Paramonov, sp. nov**.
–	Posterior process of paramere elongate, shaped like a hooked spine (Zhang and Kang 2021: fig. 4C)	***Ptychoptera yunnanica* Zhang & Kang, 2021**
4	Apices of epandrial clasper strongly curved medially, tips opposing each other (Fig. 17A, B)	**5**
–	Apices of epandrial clasper straight, at most weakly convergent medially, tips nearly parallel (Fig. 7B)	**7**
5	Tips of epandrial claspers curved dorsally (Shao and Kang 2021: fig. 3a), apex of anterior branch (B_2_) of basal lobe of gonostylus cleft (Shao and Kang 2021: fig. 3c)	***Ptychoptera tianmushana* Shao & Kang, 2021**
–	Tips of epandrial claspers not curled dorsally apex of anterior branch (B_2_) of basal lobe of gonostylus rounded	**6**
6	Wing with broken brown band from pterostigma to M fork (Kang et al. 2019: fig. 3c)	***Ptychoptera emeica* Kang, Xue & Zhang, 2019**
–	Wing with brown spot at R_4+5_ fork, sometimes faint spots at R_1+2_ and M forks, but not a coherent band (Kang et al. 2013: fig. 8)	***Ptychoptera lii* Kang, Yao & Yang, 2013**
7	Epandrial clasper with distinct posterior curve near apex	**8**
–	Epandrial clasper without posterior curve	**9**
8	Gonostylus with apex of primary branch weakly curved and terminating in a rounded knob (Fig. 11B, C), parameres with elongate spines which cross medially (Fig. 11E)	***Ptychoptera persimilis* Alexander, 1947**
–	Gonostylus with apex of primary branch hooked (Kang et al. 2013: fig. 17), parameres with triangular projections which do not cross medially (Kang et al. 2013: fig. 17)	***Ptychoptera lushuiensis* Kang, Yao & Yang, 2013**
9	Spathate lobes of hypandrium tongue-like, with rounded apex (Fig. 13A, B)	***Ptychoptera pseudosimilis* Fasbender, sp. nov**.
–	Spathate lobes triangular or blade-like, tapering	**10**
10	Gonostylus with basal portion of secondary branch rounded in lateral view (Nakamura and Saigusa 2009: fig. 23); paramere posterior process directed laterally, flattened subtriangular plate with triangular spine dorsally (Nakamura and Saigusa 2009: fig. 25)	***Ptychoptera formosensis* Alexander, 1924**
–	Gonostylus with basal portion of secondary branch subtrapezoidal dorsal margin straight (Fig. 7A, C–F); paramere posterior process directed ventromedially, sharply curved hooked projections (Fig. 7D)	***Ptychoptera annandalei* Brunetti, 1918**

### Other species

#### Ptychoptera hantu

Taxon classificationAnimaliaDipteraPtychopteridae

Kolcsár & Fasbender sp. nov.

C57A3674-F86F-58DA-80EE-58B04835D510

https://zoobank.org/262DE4CD-9F86-4010-9D38-2B8043D47ADA

Figs 14B, 19–25

##### Type material.

***Holotype*. Singapore** • male; Reservoir Park, Central Catchment NR, Sime Track; 25 m a.s.l.; 1.352611°N, 103.80597°E; 05.IX.2005; P. Grootaert leg.; ZRCENT00035821; ZRC. HOLOTYPE *Ptychoptera hantu* Kolcsár & Fasbender, sp. nov. [red label]. ***Paratypes*** (2 males, 9 females). **Singapore** • 1 male, 3 females; Reservoir Park, Central Catchment NR, Sime Track; 25 m a.s.l.; 1.352611°N, 103.80597°E; 17.VI.2005; P. Grootaert leg.; ZRCENT00035824, ZRCENT00035825, ZRCBDP0047774 (COI barcode Genbank accession code PX672981); ZRC. • 1 copula (1 male, 1 female); Reservoir Park, Central Catchment NR, Sime Track; 25 m a.s.l.; 1.352611°N, 103.80597°E; 17.VI.2005; P. Grootaert leg.; ZRCBDP0455402, ZRCBDP0455403; ZRC. • 2 females; Reservoir Park, Central Catchment NR, Sime Track; 25 m a.s.l.; 1.352611°N, 103.80597°E; 22.IV.2005; P. Grootaert leg.; ZRCENT00035822, ZRCENT00035826; ZRC. • 3 females; Reservoir Park, Central Catchment NR, Sime Track; 25 m a.s.l.; 1.352611°N, 103.80597°E; 02.VI.2005; P. Grootaert leg.; ZRCBDP0296178 (COI barcode GenBank accession code PX672982), ZRCENT00035823, ZRCENT00035827; ZRC.

##### Other observations.

**Figure 19.**
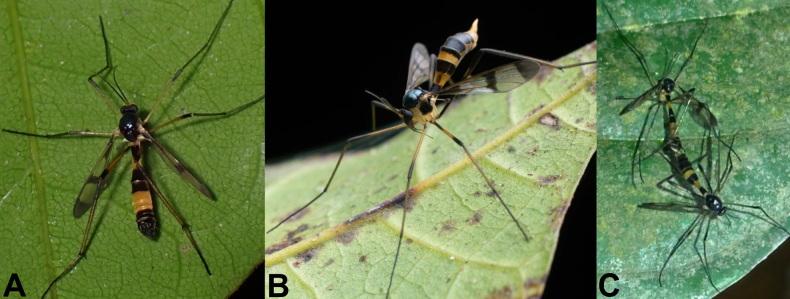
*Ptychoptera hantu* Kolcsár & Fasbender, sp. nov. **A**. Male (photo Ong Ei Leen); **B**. Female (photo Kenneth Chi); **C**. Copula (photo Benedict Yeap).

**Singapore** • 1 male; Upper Seletar Reservoir Park, Track 7, 1.39985°N, 103.80693°E; 30.X.2022; observer/photographer: Ong Ei Leen (macrocipher). https://www.inaturalist.org/observations/268917928; (Fig. 19A). • 1 female; Upper Seletar Reservoir Park, Track 7, 1.39985°N, 103.80693°E; 8.III.2022, observer/photographer: Kenneth Chin (kennethchinimages). https://www.inaturalist.org/observations/108161249; Fig. 19B. • 1 copula (1 male, 1 female); Upper Seletar Reservoir Park, Track 7, 1.39936°N, 103.80774°E; 2.X.2025; observer/photographer: Benedict Yeap (bugboiben) https://www.inaturalist.org/observations/323406074; Fig. 19C.

##### Diagnosis.

Vein Sc ending opposite of fork of Rs. Male genitalia with epandrial claspers digitiform with setose swelling subapically, gonopods with apical lobe longer than gonocoxite and directed posteroventrally.

##### Description.

**Male. *Measurements***: Body length 6.5–6.7 mm, wing length 4.8–5 mm, width 1.5–1.6 mm (*n* = 2). Antenna 3.5 mm (*n* = 1).

**Figure 20.**
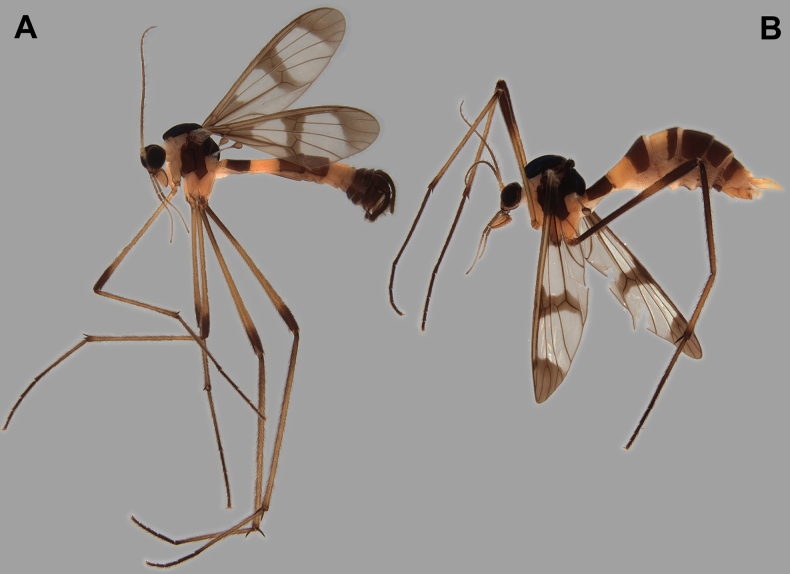
*Ptychoptera hantu* Kolcsár & Fasbender, sp. nov. Habitus. **A**. Male; **B**. Female.

***Head***. Vertex shining black, blue from certain angles, glabrous; frons extremely narrow strip brown; occiput dark brown medially, pale brown behind eyes with a dark elliptical spot (stemmatic bulla); gena very narrow brown; occiput and gena with brown setae; facial sclerite large, dark brown, yellow below the scapes; clypeus approx. hexagonal, weakly inflated, brown with anterior margin black, with few dark brown sensilla; palpus brown, last segment pale brown with base and tip narrowly brown; labrum narrowly spade or pentagonal shaped, anterior margin dark brown; labium pale brown; labellum pale brown with brown setae (Fig. 14B). Scape, pedicel yellow; basal 1/2 of first flagellomere pale brown yellow, remaining flagellum brown, setae on antenna dark brown; first flagellomere 1.8× longer than second flagellomere (Figs 14B, 20A).

**Figure 21.**
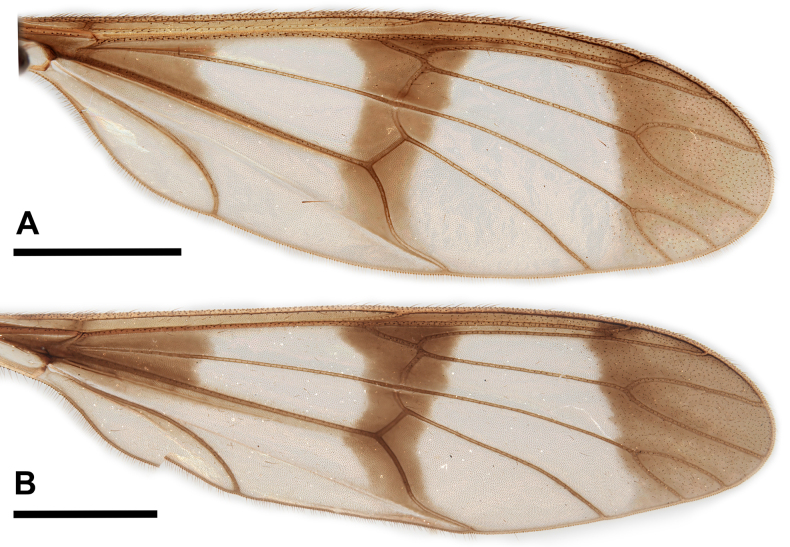
*Ptychoptera hantu* Kolcsár & Fasbender, sp. nov. Wings. **A**. Male; **B**. Female. Scale bar: 0.5 mm.

***Thorax***. Antepronotum yellow; postpronotum yellow; prescutum uniformly shiny black, blue in certain angles; scutum uniformly shiny black, blue in certain angles; scutellum dark brown, with dark brown setae; postalar callus dark brown; postalar wall dark brown; mediotergite uniformly shiny black, blue in certain angles; paratergite shiny black; propleuron yellow; anepisternal cleft yellow; anepisternum dark brown; katepisternum dark brown, anterior basalare dark brown; posterior basalare dark brown; epimeron dark brown; laterotergite dark brown with patch of long setae; metanepisternum yellow; metakatepisternum yellow; metanotum yellow (Figs 19A, 20A). ***Wing***. Wing length/width ratio 3.2. Three brown pigment bands: basal band brown, from h to 1/2 of length R, between R and CuA; middle band present, brown, around from fork of Rs to proximal angle of CuA apical band reaching wing apex, extending from pterostigma to posterior margin beyond M2, rarely m1 and/or m2 with transparent patch; cells c and sc brown; pterostigma brown. Membrane with microtrichia around fork of Rs, c after tip of Sc, and in apical band. Sc terminates just short of r-m; apex of R1 slightly before or at fork of R_4+5_; Rs subequal to r-m; r-m joins R_4+5_ distal to R3 fork; R4 2/3 length of R_4+5_, proximal curve of R4 even arc. M_1+2_ fork 2/3 length of R_4+5_ fork. CuA sigmoidal distal to m-cu (Fig. 21A). ***Halter***. Stem basal 1/3 white or yellow, apical 2/3 pale brown; knob brown. Prehalter yellow (Fig. 20A). ***Legs***. Fore coxa yellow; fore trochanter pale brown; fore femur pale brown, apical 1/5 dark brown; fore tibia pale brown; fore tarsomere 1 pale brown, apical 1/6 dark brown; fore tarsomeres 2–5 dark brown; mid coxa yellow; mid trochanter pale brown; mid femur pale brown, apical 1/5 dark brown; mid tibia pale brown; mid tarsomere 1 pale brown, apical 1/2 dark brown; mid tarsomeres 2–5 dark brown; hind coxa yellow; hind trochanter pale brown; hind femur basal 1/3–1/2 brown, middle pale brown, apical 1/6 dark brown; hind tibia basal 1/5 brown, apical 4/5 pale brown; hind tarsomere 1 pale brown, apical 1/5 dark brown; hind tarsomeres 2–5 dark brown (Figs 19A, 20A).

***Abdomen***. Tergite 1 anterior 1/4 yellow, posterior 3/4 dark brown; tergite 2 anterior 1/2 yellow, posterior 1/2 dark brown; tergite 3 dark brown; tergite 4 yellow; tergite 5 yellow, with or without small middle brown patch; tergite 6 dark brown; tergite 7 dark brown; sternite 1 yellow; sternite 2 anterior 1/2 yellow, posterior 1/2 brown; sternite 3 brown, without accessory copulatory organ; sternite 4 yellow; sternite 5 yellow; sternite 6 dark brown; sternite 7 dark brown (Figs 19A, 20A).

**Figure 22.**
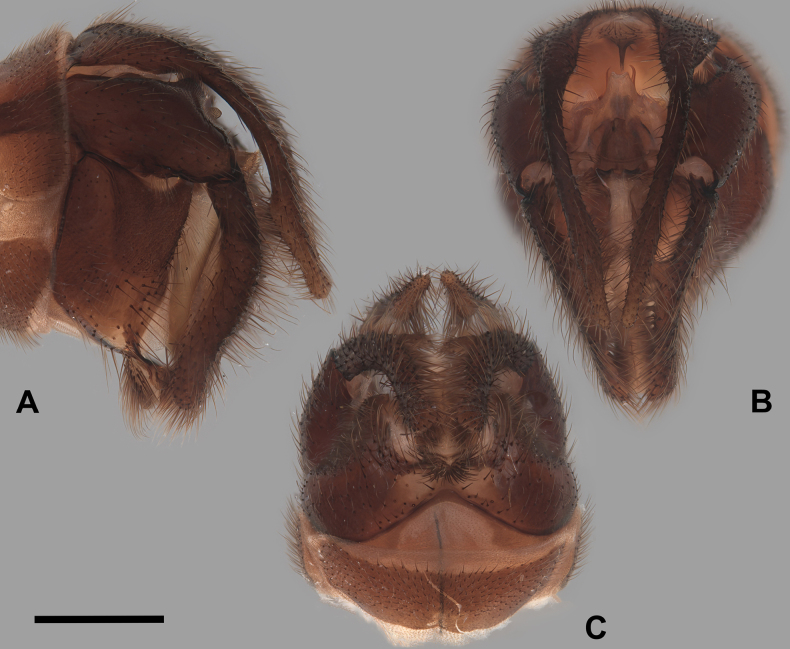
*Ptychoptera hantu* Kolcsár & Fasbender, sp. nov. Unmacerated male terminalia. **A**. Lateral; **B**. Posterior; **C**. Ventral. Scale bar: 0.5 mm.

**Figure 23.**
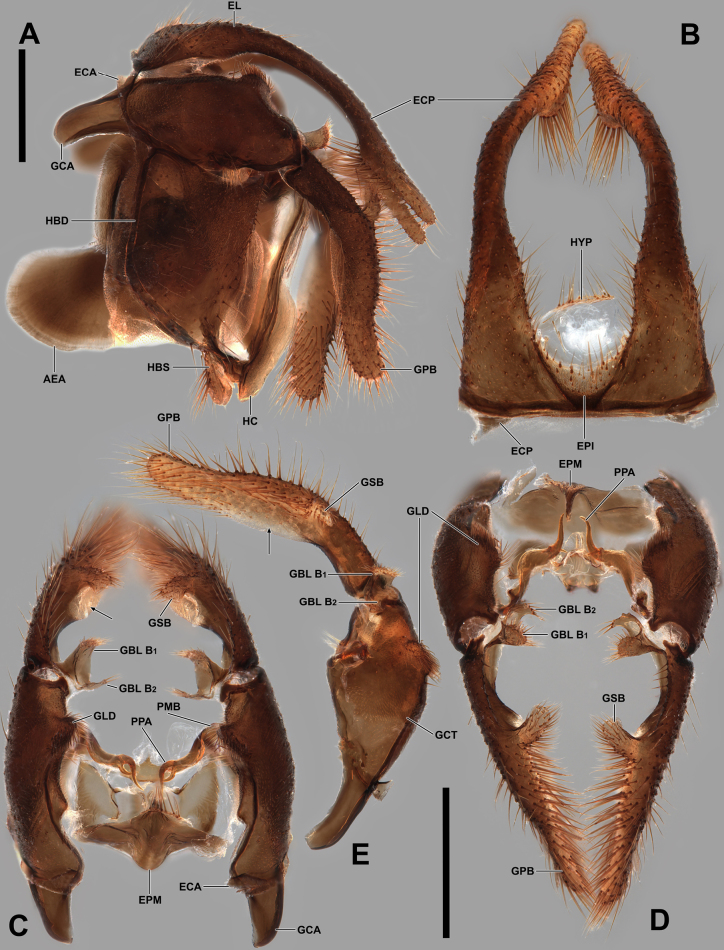
*Ptychoptera hantu* Kolcsár & Fasbender, sp. nov. **A**. Overall, lateral; **B**. Epandrium, dorsal; **C**. Gonopods and parameres, dorsal; **D**. Gonopods and parameres, posterior; **E**. Gonopod, inner lateral. Scale bar: 0.5 mm.

***Male terminalia***. Dark brown (Fig. 22). Epandrial collar complete. Epandrial apodemes well developed, thread-like, extending across gonocoxal apodeme to hypandrium, with breaks present at epandrium and hypandrium (Fig. 23A). Epandrial lobes digitiform, elongate, narrow (Fig. 23B). Contiguous epandrial clasper indistinguishable from epandrial lobe, strongly curved ventrally without distinct angle; apex rounded, with prominent medial swelling at 3/5 length of epandrial lobe, narrowing toward apex, bearing prominent long setae, longest at base of swelling (Figs 22, 23A, B). Epiproct readily apparent, posterior margin of epiproct U-shaped, completely separating epandrial lobes (Fig. 23B). Hypoproct fused to subepandrial sclerite, border indistinguishable (Figs 22B, 23B–D). Subepandrial membrane fully sclerotized, forming a complex invagination with equal-length dorsal and ventral sclerites connected medially by a narrow bridge; dorsal sclerite with a curved finger-like lobe below hypoproct, semicircular ridge medially (Figs 22B, 23B–D); ventral sclerite semicircular, with a pair of lateral longitudinal dorsal ridges and a median triangular ridge below parameres (Fig. 23C); lateral sclerites not separated from ventral sclerite. Parameres clearly distinguishable, connected basally at apical lobe by a less sclerotized, flat, plate-like bridge, posterior margin ending in a sharp point (Fig. 23C, D). Parameres arched and twisted inward in dorsal view (Fig. 23C), directed dorsally in posterior view (Fig. 23D); apical process with a sinuous acute spine directed dorsomedially and a subapical acute spine directed inward (Fig. 23C, D). Gonocoxal apodeme tongue-shaped, curved ventrally, 1/2 the length of gonocoxite; dorsal spur minute, rounded, longer than tall (Fig. 23E). Gonocoxite approx. lentiform, dorsal and ventral margins convex (Fig. 23A, E). Dorsal gonocoxal lobe present, rectangular, covered with a patch of short, dense setae (Figs 22A, 23A, 23C–E). Basal lobe of gonostylus divided into two branches: B_1_ roughly triangular, directed inward, with few pale setae; B_2_, narrow, finger-like, directed inward with few setae; B_1_ and B_2_ forming a semicircular cavity (Fig. 23C, D).

**Figure 24.**
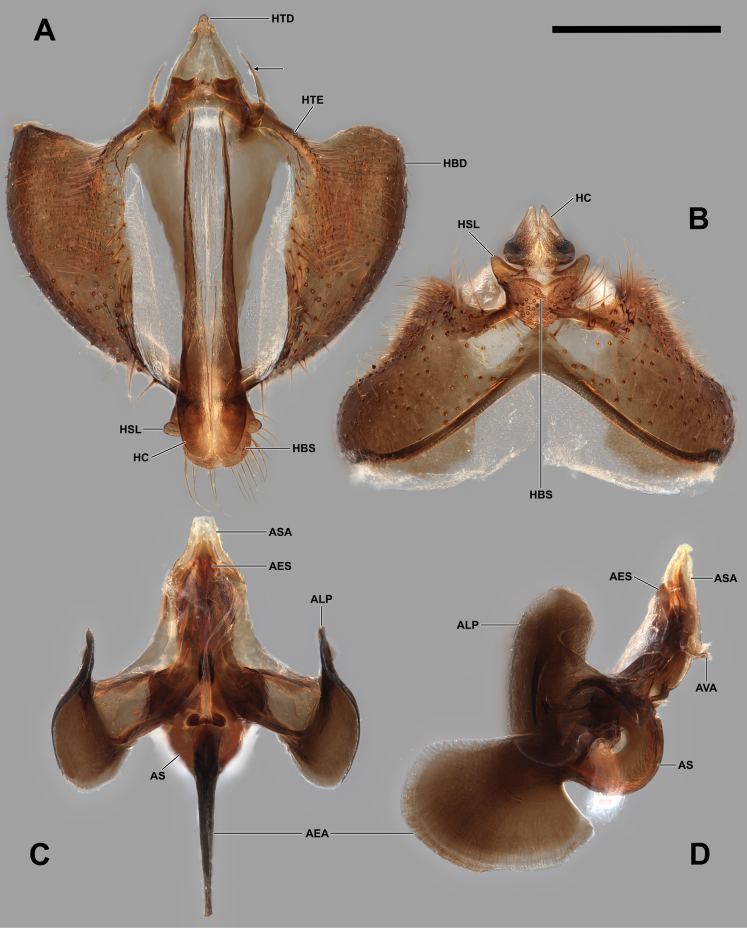
*Ptychoptera hantu* Kolcsár & Fasbender, sp. nov. Male terminalia. **A**. Hypandrium, posterior; **B**. Hypandrium, ventral; **C**. Aedeagus, anterior; **D**. Aedeagus, lateral. Scale bar: 0.5 mm.

Primary branch very long, strongly directed ventrally, basally cylindrical, laterally flattened beyond the secondary branch, with membranous swelling (Fig. 23C, E, arrow). Secondary branch present, finger-like, directed inward in dorsal view (Fig. 23C) and slightly curving anteriorly in posterior view (Fig. 23D); both primary and secondary branches covered with long brown setae. Hypandrium with basal division approx. rectangular in lateral view (Figs 22A, 23A); basal scale present, small, undivided, cordate (Fig. 24A, B). Spathate lobe present, very small, bare, directed ventrally and curving posteriorly in lateral view (Fig. 23A). Hypandrial conjunctive present, reaching the base of the terminal division (Fig. 24A), ventral end rounded, in cross-section resembling a crane rail (Fig. 24B). Terminal division with well-developed lateral extensions connected to basal division (Fig. 24A); terminal division apex medially triangular, pointed, with two basal filiform lobes, directed posteriorly (Fig. 24A, arrow). Aedeagus tilted 45 degrees from vertical in lateral view (Fig. Fig. 24D). Aedeagal sclerite weakly convergent, rounded apically in dorsal view (Fig. 24C), subequal in length to sperm sac; apical apodemes present. Lateral ejaculatory processes with discoid apodeme reniform, extending dorsally from base (Fig. 24C, D). Sperm sac subspherical, smaller than ejaculatory apodeme in lateral view (Fig. 24D). Ejaculatory apodeme fan shaped, not adjacent to base of aedeagus, extending anteriorly into segment VII (Figs 23A, 24C). Subaedeagal sclerite flattened, partly membranous, apex subequilateral triangle in anterodorsal view (Fig. 24C), ventral arm of subaedeagal sclerite present, acute spine weakly curved medially (Fig. 24D).

**Female. *Measurements***: Body length 7.1–8.3 mm, wing length 5.8–6.7 mm, width 1.5–1.9 mm (*n* = 6) Antenna 2.2–3 mm (*n* = 4).

***Head***. As in male. First flagellomere 2–2.3× longer than second flagellomere.

***Thorax***. As in male. ***Wing and halter***. As in male. Wing length/width ratio 3.4–3.7. ***Legs***. Differ from male in following details: Fore femur basal 2/3 pale brown, apical 1/3 dark brown; fore tibia pale brown, basally dark brown in some specimens; fore tarsomere 1 pale brown to dark brown; fore tarsomeres 2–5 dark brown. Mid femur basal 1/4 brown, middle pale brown, apical 1/4 dark brown; mid tibia basal 1/6 brown, middle pale brown, apical 1/6 dark brown; mid tarsomere 1 dark brown; mid tarsomeres 2–5 dark brown. Hind femur basal 3/5 dark brown, 1/5 pale brown, apical 1/5 dark brown; hind tibia basal 1/2 dark brown, apical 1/2 pale brown, extreme tip dark brown; hind tarsomere 1 basal 1/2 pale brown, apical 1/2 dark brown; hind tarsomeres 2–5 dark brown (Figs 19B, 20B).

***Abdomen***. Tergite 1 anterior 1/5 yellow, posterior 4/5 dark brown; tergite 2 anterior 1/2 yellow, posterior 1/2 dark brown; tergite 3 anterior 1/3 yellow, posterior 2/3 dark brown; tergite 4 dark brown; tergite 5 dark brown; tergite 6 dark brown; tergite 7 dark yellow; sternite 1 yellow; sternite 2 yellow or pale brown; sternite 3 yellow; sternite 4 brown; sternite 5 dark brown; sternite 6 dark brown; sternite 7 dark yellow (Figs 19B, 20B).

**Figure 25.**
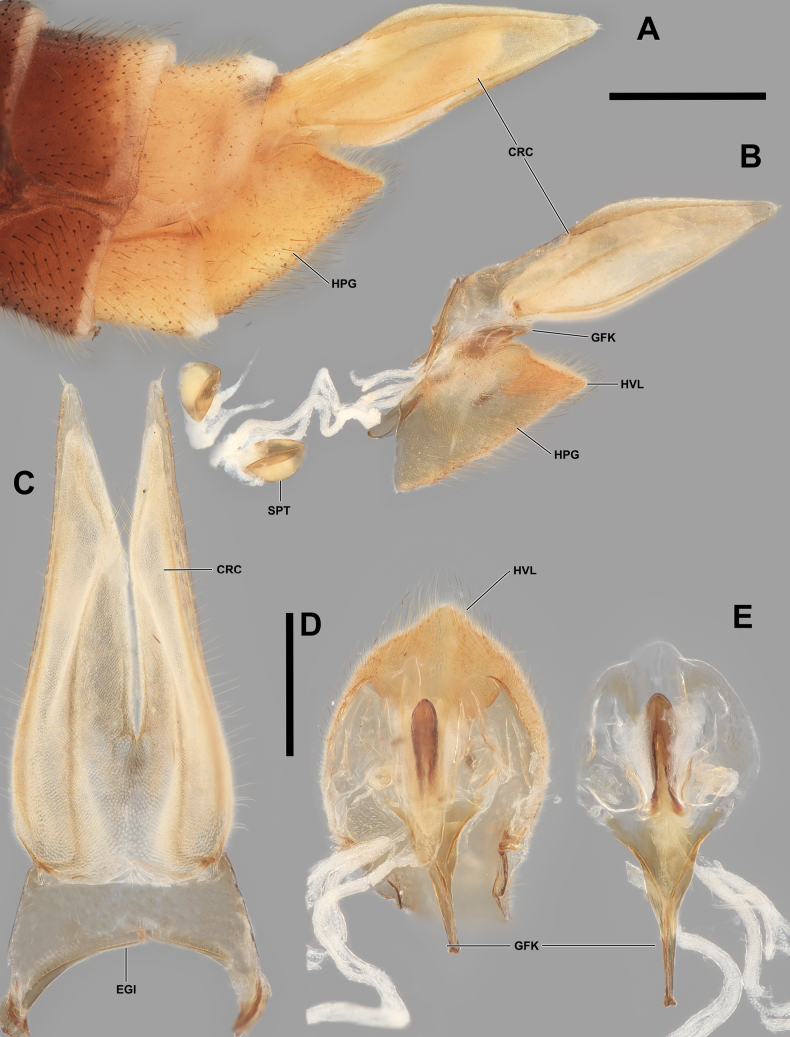
*Ptychoptera hantu* Kolcsár & Fasbender, sp. nov. Female terminalia. **A**. Unmacerated, lateral; **B**. Macerated, lateral (one spermathecae missing); **C**. Epigynium and cerci, ventral; **D**. Hypogynium, hypogynial valves and genital plate, dorsal; **E**. Genital plate. Scale bars: 0.5 mm (**A, B**); 0.25 mm (**C–E**).

***Female terminalia***. Hypogynium without a lobe at the corner (Fig. 25A, B). Separation between the hypogynium and hypogynial valves indistinct; apex rounded, without incision (Fig. 25D). Cerci grain-shaped. Sternite 9 with the posterior margin forming a rounded plate bearing a distinct median rounded lobe. Bursa dark, finger-shaped, with two rounded lateral invaginations (Fig. 25D, E). Three spermathecae, relatively large, spherical, ~ 2/3 width of the cerci in lateral view (Fig. 25B).

##### Distribution.

Singapore.

##### Etymology.

The species epithet *hantu* is the Malay word for ghost, referring to the common English name “phantom crane flies,” as well as to the species’ hidden lifestyle in the swamp forests of Singapore. The epithet is a noun in apposition.

##### Habitat.

Sime Track is situated within Singapore’s Central Catchment Nature Reserve (CCNR), the nation’s most ecologically rich forest–stream systems. Its shaded headwater and blackwater habitats with organic-rich soils and stable humidity support exceptionally high yet largely undocumented insect diversity, representing some of the most important refugia for undiscovered species in Singapore.

##### Remarks.

*Ptychoptera hantu* does not belong to any currently recognized species group of *Ptychoptera*. The very small, bare spathate lobes differentiates it from most of the other southeast Asian *Ptychoptera*. *Ptychoptera hantu* is readily recognized among the Indomalayan *Ptychoptera* by the combination of ventrally directed apices of the gonostylus and aforementioned small and bare spathate lobes.

#### Ptychoptera perbona

Taxon classificationAnimaliaDipteraPtychopteridae

Alexander, 1946

79C0445A-925D-5E5A-B5C3-95C59DBE7CBC

Figs 2B, 26–33

Ptychoptera perbona flaviventris Alexander, 1946, syn. nov.

##### Type material examined.

***Ptychoptera perbona flaviventris*: *Holotype*. Myanmar (Burma)** • male, N.E. Burma, Kambaiti, 7000 m, 12.VI.1934; R. Malaise leg.; USNM (slide).

***Ptychoptera perbona*: *Paratype*. Myanmar (Burma)** • male, N.E. Burma, Kambaiti, 7000 m, 16.V–16.VI.1934; R. Malaise leg.; USNM (slide).

##### Additional material.

**Thailand** • 1 male; Chiang Mai, Chomthong, Doi Inthanon NP, Check point 2; 1639 m a.s.l.; 18.527867°N, 98.499876°E; 29.V–1.VII.2014; Malaise trap 1; W. Srisuka R. Sawkord T. Somboonchai, S. Suriya leg.; QSBG. • 1 male; Tak, Umphang, Umphang Wildlife Sanctuary, Mae klong keer anger unit; 1234 m a.s.l.; 16.225548, 98.980368; 2–28.IV.2020; Malaise trap 2; J. Chomrit leg., QSBG. **Vietnam** • 1 male; Lai Châu Province, Hoàng Liên NP, 22.34997°N, 103.76818°E, 1947 m. 11.IV.2012; leg. D.I. Gavryushin. • as previous except 3 males; 16.IV.2012; ZISP.

##### Diagnosis.

Male genitalia with epandrial claspers having ventromedial curve at apex. Gonostylus with basal lobe split into two lobules branch, primary and secondary branch of apical lobe weakly divided. Hypandrium without basal scale and spathate lobes.

##### Redescription.

Both the body coloration and male genitalia show significant differences among specimens. However, since we have access to only a limited number of specimens from each population, it is not possible to conclude whether these differences represent distinct species, subspecies, or simply natural variation within the species (see Discussion). The general species description is based on specimens collected in Thailand and Vietnam.

**Male. *Measurements*** (including Alexander 1946): Body length 7–7.5 mm, wing length 7.1–8.5 mm, width 1.9 mm (*n* = 1). Antenna 4.2–4.8 mm.

**Figure 26.**
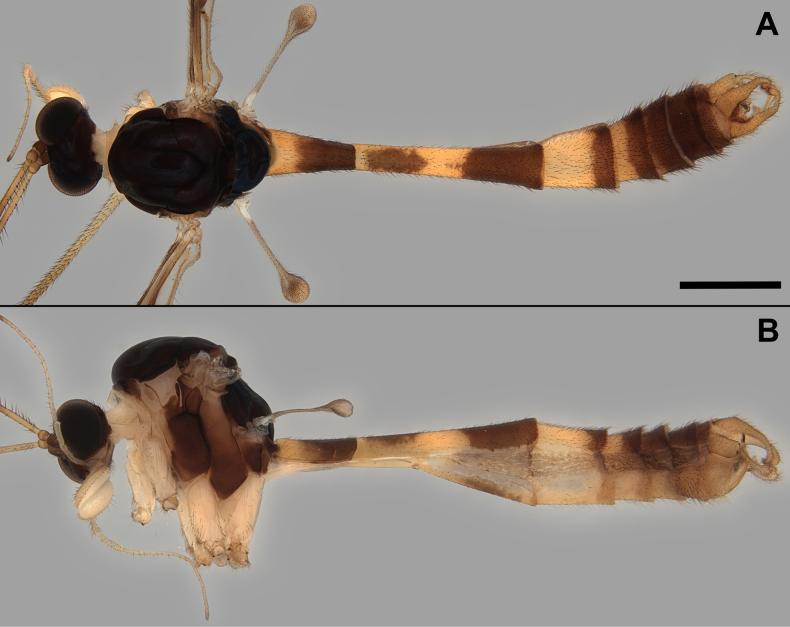
*Ptychoptera perbona* Alexander, 1946. Male habitus. **A**. Dorsal; **B**. Lateral. Scale bar: 1 mm.

***Head***. Vertex shining black, blue from certain angles, glabrous; frons narrow strip dark brown; occiput uniformly dark brown, stemmatic bulla with black spot; gena narrow dark brown; occiput and gena with sparse elongate dark brown setae; facial sclerite weakly delineated from clypeus, dark brown; clypeus rhomboidal, inflated, brown, covered with dark brown setae; palpus pale brown, last segment, tip dark brown; labrum pentagonal or slightly shade shaped; labium yellow; labellum yellow with pale brown setae. Scape dark brown; pedicel brown; first flagellomere pale brown basal 1/2 pale brown, apical 1/2 brown; remaining flagellum brown to dark brown. First flagellomere 1.4–1.7× longer than second flagellomere (Figs 2B, 26).

**Figure 27.**
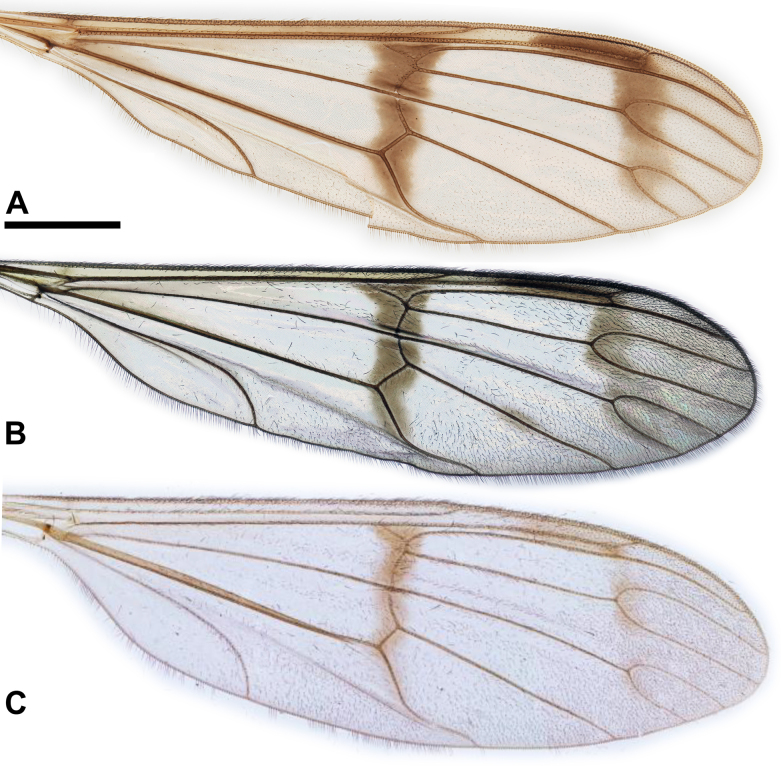
*Ptychoptera perbona* Alexander, 1946. Wings. **A**. Specimen from Thailand: Tak, Umphang Wildlife Sanctuary, 16.225548, 98.980368. **B**. Specimen from Vietnam: Lai Châu Province, Hoàng Liên NP, 22.34997°N, 31.0376818°E. **C**. *Ptychoptera perbona flaviventris* holotype. Scale bar: 0.5 mm.

***Thorax***. Antepronotum yellow; postpronotum brown; prescutum uniformly shiny black, blue in certain angles; scutum uniformly shiny black, blue in certain angles; scutellum dark brown, with round poorly visible brown spot in middle with dark brown setae; postalar callus dark brown; postalar wall dark brown; mediotergite black, blue in certain angles; paratergite black; propleuron yellow to pale brown; anepisternal cleft yellow; anepisternum brown; katepisternum brown; anterior basalare brown; posterior basalare brown; epimeron uniformly brown or pale brown to brown, ventrally always darker, degree of coloration variable; laterotergite shiny black with patch of long setae; metanepisternum pale brown; metakatepisternum brown; metanotum pale brown to brown (Fig. 26). ***Wing***. Wing length/width ratio 3.2–3.8. Two brown pigment bands: basal band absent; middle band from fork of Rs to proximal angle of CuA; subapical band not reaching wing apex, between pterostigma and M fork; cells c pale brown, sc pale brown, transparent between apex of Sc and pterostigma, pterostigma brown (Fig. 27A, B). Membrane with microtrichia in every cell, less abundant in br, bm, cua, cup. Sc terminates 2× length of r-m beyond fork of Rs; apex of R1 beyond fork of R_4+5_; Rs subequal to r-m; r-m joins R_4+5_ distal to R3 fork, R4 three quarters length of R_4+5_; proximal curve of R4 even arc. M_1+2_ fork 2/3 length of R_4+5_ fork. CuA sigmoidal distal to m-cu (Fig. 27). ***Halter***. Stem basal 1/3 white or yellow, apical 2/3 pale brown; knob brown. Prehalter brown (Fig. 26B). ***Legs***. Fore coxa yellow; fore trochanter pale brown; fore tibia deepening to brown apically, apical 1/5–1/6 dark brown; fore femur pale brown deepening to brown apically, or apical 1/6 dark brown; fore tarsomere 1 brown; fore tarsomeres 2–5 dark brown. Mid coxa yellow; mid trochanter pale brown; mid femur pale brown apical 1/7 dark brown; mid tibia apical 1/7 dark brown; mid tarsomere 1 brown deepening to dark brown; mid tarsomeres 2–5 dark brown. Hind coxa yellow; hind trochanter pale brown; hind femur pale brown, apical 1/7 dark brown; hind tibia pale brown apical 1/7 dark brown; hind tarsomere 1 pale brown pale brown deepening to brown; hind tarsomeres 2–5 dark brown.

***Abdomen***. Greatly variable in coloration. Tergite 1 uniformly black or anterior 1/3 yellow, posterior 3/4 dark brown; tergite 2 dark brown with two yellow area either side at near midlength or anterior 1/2 yellow with a basal brown patch, posterior 1/2 dark brown; tergite 3 anterior 1/2–2/3 yellow, posterior 1/2–1/3 brown; tergite 4 uniformly dark brown or anterior 1/2 yellow, posterior 1/2 brown; tergite 5 uniformly dark brown or anterior 1/3 yellow, posterior 2/3 brown; tergite 6 brown; tergite 7 brown to dark brown. Sternite 1 yellow; sternite 2 anterior 1/2 yellow, posterior 1/2 pale brown; sternite 3 yellow, without accessory copulatory organ; sternite 4 pale brown; sternite 5 pale brown; sternite 6 pale brown; sternite 7 pale brown (Fig. 26).

**Figure 28.**
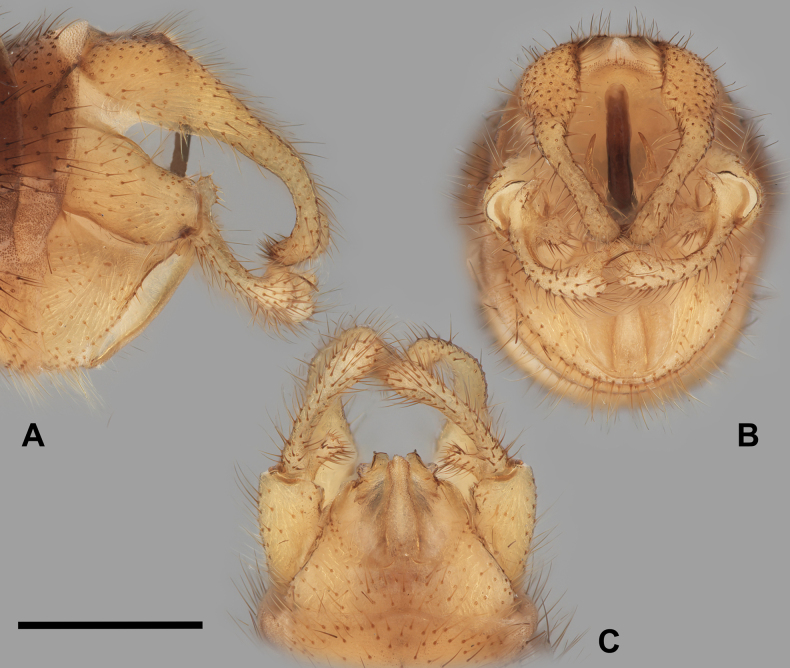
*Ptychoptera perbona* Alexander, 1946. Unmacerated male terminalia. **A**. Lateral; **B**. Posterior; **C**. Ventral. Scale bar: 0.5 mm. Specimen from Thailand: Tak, Umphang Wildlife Sanctuary, 16.225548, 98.980368.

**Figure 29.**
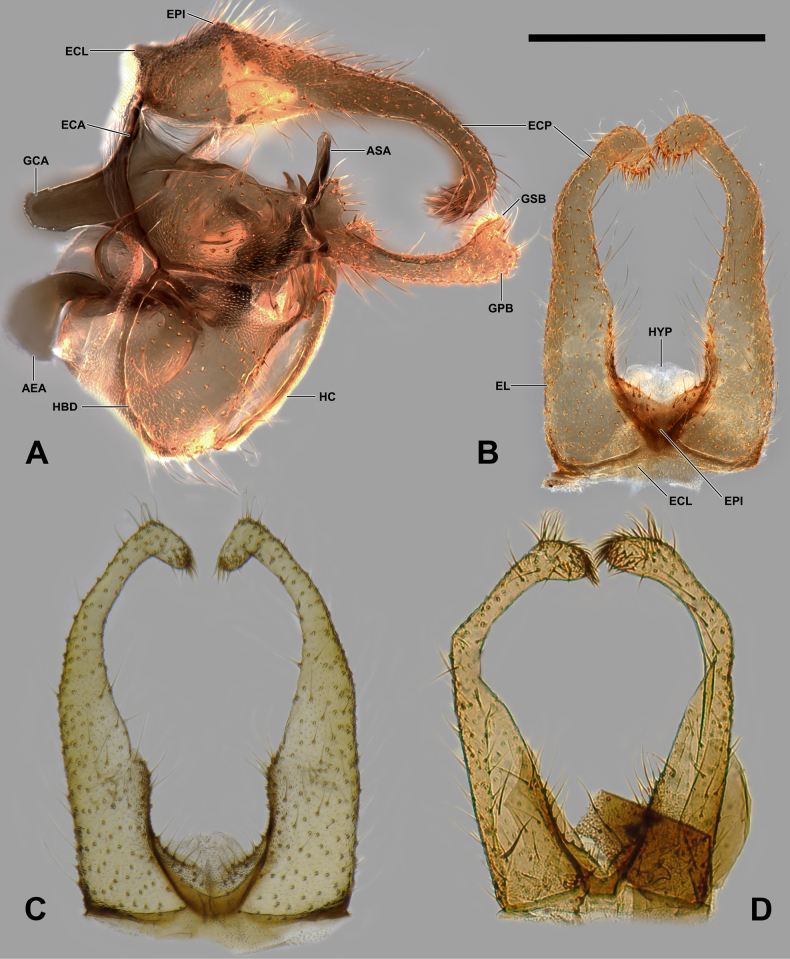
*Ptychoptera perbona* Alexander, 1946. **A**. Overall, lateral; **B–D**. Epandrium, dorsal. **A, B**. Specimen from Thailand: Tak, Umphang Wildlife Sanctuary, 16.225548, 98.980368. **C**. Specimen from Vietnam: Lai Châu Province, Hoàng Liên NP, 22.34997°N, 31.0376818°E. **D**. *Ptychoptera perbona perbona* paratype, Myanmar: Kambaiti Pass. Scale bar: 0.5 mm.

**Figure 30.**
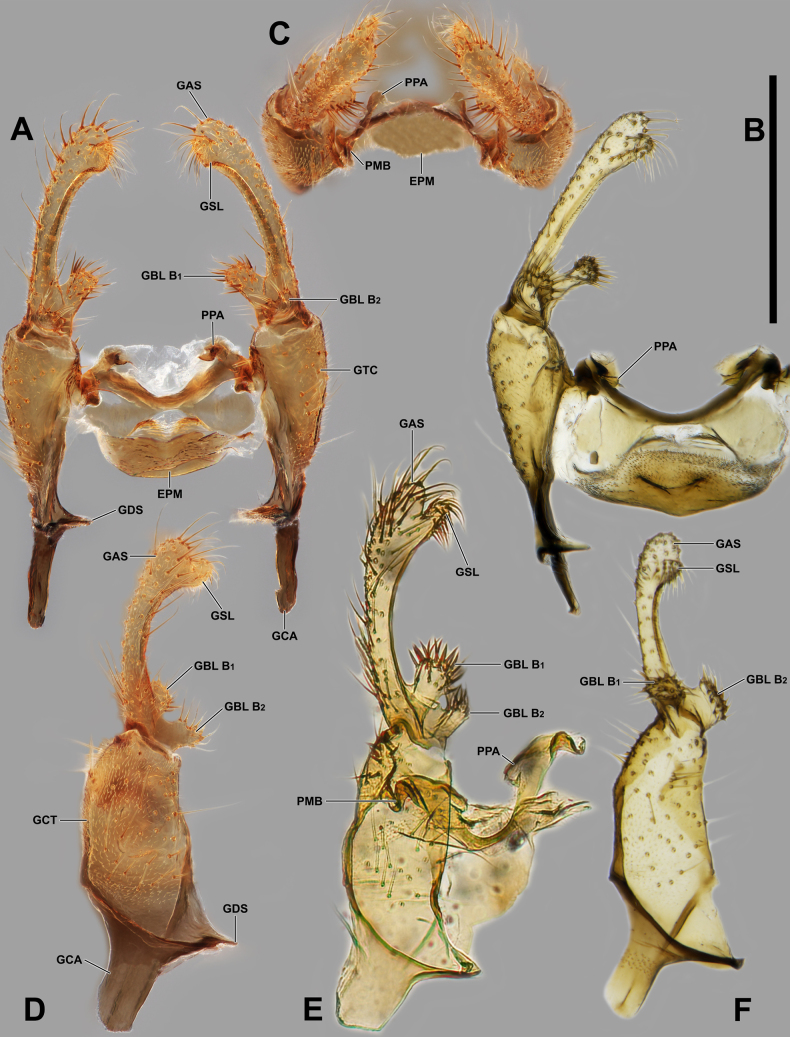
*Ptychoptera perbona* Alexander, 1946. **A, B, E**. Gonopods, parameres and subepandrial sclerite, dorsal; **C**. Gonopods and parameres, posterior; **D**. Gonopod, outer lateral; **F**. Gonopod, inner lateral. **A, C, D**. Specimen from Thailand: Tak, Umphang Wildlife Sanctuary, 16.225548, 98.980368. **B, F**. Specimen from Vietnam: Lai Châu Province, Hoàng Liên NP, 22.34997°N, 31.0376818°E. **E**. *Ptychoptera perbona perbona* paratype, Myanmar: Kambaiti Pass. Scale bar: 0.5 mm.

***Male terminalia***. Pale brown to brown (Fig. 28). Epandrial collar complete (Fig. 29A). Epandrial apodemes well developed, thread-like, extending across gonocoxal apodeme to hypandrium, with break only present at epandrium (Fig. 29A). Epandrial lobes digitiform, elongate (Fig. 29). Contiguous epandrial clasper indistinguishable from epandrial lobe, narrow, gently curved inward; apex rounded, slightly bulbous, curved ventrally and inward, with a patch of short black setae (Fig. 29). Epiproct triangular, reaching anterior margin of epandrium; posterior margin of epiproct V-shaped, completely separating epandrial lobes (Fig. 29C, D). Hypoproct small sclerite, fused with subepandrial sclerite, partly membranous (Fig. 30A). Subepandrial membrane fully sclerotized, large; anterior-dorsal sclerite larger than ventral-posterior sclerite; lateral sclerites fused with ventral sclerite (Fig. 30A, B). Parameral bridge undivided, directed inward after a short distance, arcing dorsally; distal margin convex, shape variable; apical process small and slightly rounded, anterior margin more strongly sclerotized; lobe directed inward in dorsal view (Fig. 30A, B) and dorsally in lateral and posterior view (Fig. 30C). Gonocoxal apodeme weakly tapering, apex rounded, 1/2 length of gonocoxite, width variable, ranging from half to nearly equal to that of the gonocoxite (Fig. 30D–F). Dorsal spur of gonocoxal apodeme present, shifted posteriorly onto anterodorsal portion of gonocoxite, about 1/2 the size of gonocoxal apodeme, acute (Fig. 30D–F). Gonocoxite subcylindrical. Gonocoxal lobes absent; a few short dark brown setae present on ventromedial margin between base of gonostylus and base of parameres. Gonostylus with basal lobe divided into two branches: both posterior branch (B_1_) and anterior branch (B_2_) approx. rectangular, with serrate edge, covered with sparse long setae.

**Figure 31.**
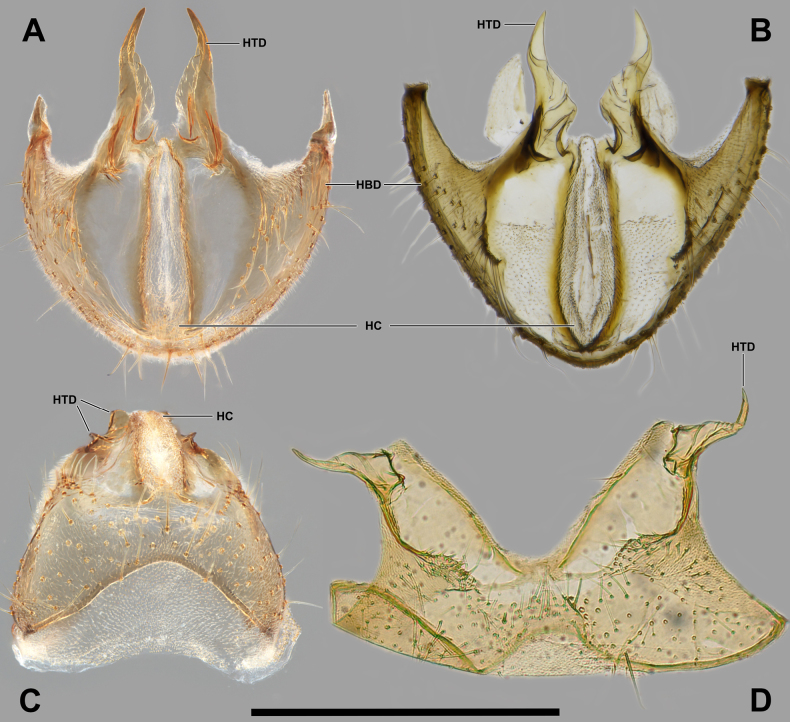
*Ptychoptera perbona* Alexander, 1946. Male terminalia. **A, B**. Hypandrium, posterior; **C**. Hypandrium, ventral; **D**. Hypandrium, slide. **A, C**. Specimen from Thailand: Tak, Umphang Wildlife Sanctuary, 16.225548, 98.980368. **B**. Specimen from Vietnam: Lai Châu Province, Hoàng Liên NP, 22.34997°N, 103.76818°E. **D**. *Ptychoptera perbona perbona* paratype, Myanmar: Kambaiti Pass. Scale bar: 0.5 mm.

**Figure 32.**
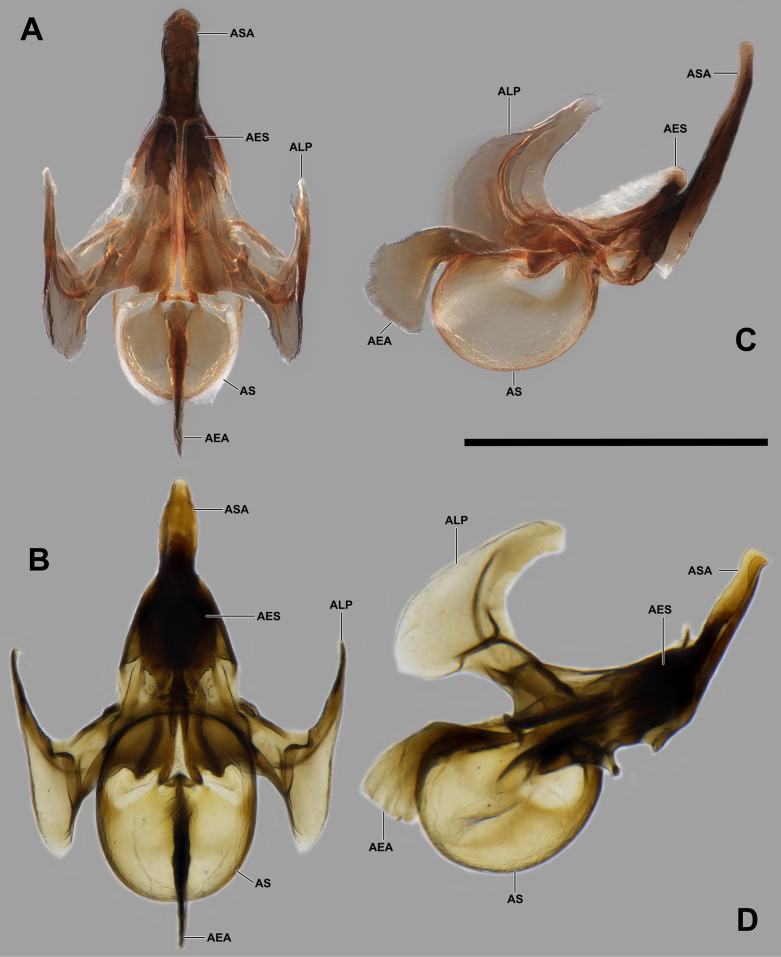
*Ptychoptera perbona* Alexander, 1946. **A, B**. Aedeagus, anterior; **C, D**. Aedeagus, lateral (**D**, slightly rotated). **A**, **C**. Specimen from Thailand: Tak, Umphang Wildlife Sanctuary, 16.225548, 98.980368. **B, D**. Specimen from Vietnam: Lai Châu Province, Hoàng Liên NP, 22.34997°N, 103.76818°E. Scale bar: 0.5 mm.

Apical lobe finger-like with a low ridge on the inner-dorsal surface, weakly laterally compressed, slightly curving dorsally, primary and secondary branches weakly separated, degree of bifurcation variable, secondary branch smaller than primary branch, and covered with a patch of setae (Fig. 30). Hypandrium with basal division sub-hemispherical (Figs 28A, 29A); spathate lobes absent; basal scale absent. Hypandrial conjunctive present, reaching base of terminal division (Figs 28A, 28B, 29A, 31). Terminal division with well-developed lateral extensions connected to basal division; apex medially divided, triangular, directed dorsally in lateral view, margin sinuate; apex elongate, pointed, slightly curved inward, shape very variable among specimens (Fig. 31A, B, D). Aedeagus tilted 20 degrees from vertical in lateral view (Fig. 32C, D), aedeagal sclerite tapering, lateral margins straight in dorsal view (Fig. 32A, B), ~ 2/3 length of sperm sac; apical apodemes present; lateral ejaculatory processes with distinct anterior angle, discoid apodeme triangular, slightly larger than ejaculatory apodeme in lateral view (Fig. 32C, D). Sperm sac ovoid, larger than ejaculatory apodeme in lateral view (Fig. 32C, D). Ejaculatory apodeme broom shaped, curved ventrally around anterior margin of sperm sac, apex broad, extending anteriorly into segment VII in lateral view (Fig. 29A), base attached anteriorly to sperm sac, not adjacent to base of aedeagus (Fig. 32C, D). Subaedeagal sclerite length variable, but in general longer than aedeagal sclerite, parallel sided rod with rounded tip in dorsal view (Fig. 32A, B); tip rectangular in dorsal view (Fig. 32C, D).

**Female. *Measurements*** (after Alexander 1946): Body length 8.5 mm, wing length 9 mm.

##### Distribution.

Myanmar/Burma (Kachin State), Thailand (northern Thailand), Vietnam (northwest).

##### Remarks.

**Figure 33.**
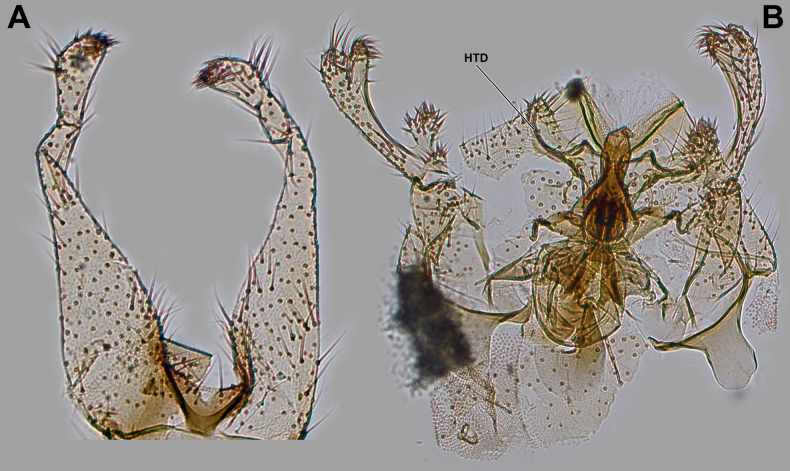
*Ptychoptera perbona flaviventris* Alexander, 1946. Male terminalia, paratype. **A**. Epandrium; **B**. Gonopods, aedeagus and part of hypandrium.

*Ptychoptera perbona* is poorly understood: the paratype of *P. perbona perbona* examined in Fasbender (2014) consisting only of badly crushed male genitalia and a wing. In the original description of *P. perbona*, Alexander (1946) described an additional subspecies, *Ptychoptera perbona flaviventris* Alexander, 1946, mainly based on differences abdominal coloration and the wing pattern being paler. The only structural difference was the supposed lack of a pair of spines medially on the terminal division in *P. perbona flaviventris*. However, the slide-mounted paratype of this subspecies (paratype not mentioned in original description but labeled) has these lobes present (Fig. 33B), though they are poorly visible and difficult to distinguish from other parts of the hypandrium due to overlapping structures. As far as can be seen, they differ in shape – the elongated apex is narrower and the basal part less wide – compared to the paratype of *P. perbona perbona* and to the Thai and Vietnamese material figured here (see Fig. 32). However, the latter specimens all show considerable variation in this structure.

Body coloration also varies among specimens, even within the type series, as already highlighted in the original description of the species. The fact that the nominotypical subspecies and *P. p. flaviventris* were collected from the same locality and time period, and that Alexander did not recognize the pair of spines on the terminal division of the hypandrium – which with color he emphasized as the main difference between the two subspecies – suggests that *P. p. flaviventris* represents intraspecific variation. The second author tried to examine the holotypes in the early 2010’s but was unable to locate them. Therefore, based on the paratype material we were able to examine, we synonymize *P. perbona flaviventris* with the nominotypical subspecies.

Currently, we have had the opportunity to study only a limited number of specimens, making it difficult to determine whether the Thai and Vietnamese specimens represent distinct subspecies or species, or whether the observed variation falls within the general variability of the species.

#### Ptychoptera phutphi

Taxon classificationAnimaliaDipteraPtychopteridae

Kolcsár & Fasbender sp. nov.

DEF17C3D-34B8-5D79-B243-41F4A5608428

https://zoobank.org/AD8730AB-3530-440E-9E22-A4A7268A09C6

Figs 2C, 3B, 34–38

##### Type material.

***Holotype*. Thailand** • male; Tak, Umphang, Umphang Wildlife Sanctuary, Mae klong kee ranger unit; 1234 m a.s.l.; 16.225548, 98.980368; 2.IV–1.V.2021; Malaise trap 2; W. Srisuka leg.; QSBG. HOLOTYPE *Ptychoptera phutphi* Kolcsár and Fasbender, sp. nov. [red label]. ***Paratypes*** (2 males). **Thailand** • 1 male; Tak, Umphang, Umphang Wildlife Sanctuary, Mae klong kee ranger unit; 1234 m a.s.l.; 16.225548, 98.980368; 2.IV–1.V.2021; Malaise trap 2; W. Srisuka leg.; LPK0001; QSBG. • 1 male; Tak, Umphang, Umphang Wildlife Sanctuary, Mae klong kee ranger unit; 1234 m a.s.l.; 16.225548, 98.980368; 2–28.IV.2020; Malaise trap 2; J. Chomrit leg.; LPK0002; QSBG.

##### Diagnosis.

Male genitalia with digitiform epandrial claspers curved ventro-anteriorly, apex T-shaped; apical stylus of gonostylus curved dorsally midway along length.

##### Description.

**Male. *Measurements***: Body length 7.6–7.9 mm, wing length 6.4–6.9 mm, width 1.7–1.8 mm (*n* = 3). Antenna ~ 3 mm (*n* = 1).

***Head***. Vertex shining dark brown to black, blue from certain angles, middle glabrous, few setae near eyes; frons extremely narrow strip brown; occiput dark brown, behind eyes yellow, glabrous, stemmatic bulla prominent without spot; gena very narrow, yellow; occiput and gena with sparse elongate brown setae; facial sclerite weakly delineated from clypeus, pale brown; clypeus rhomboidal, inflated, yellow, anterior 1/4 dark brown, covered in dark brown setae; palpus pale brown, tip of last segment brown; labrum oval, transparent with anterior margin round, black; labium yellow, labellum pale brown; labellum with brown setae. Scape pale brown; pedicel pale brown; first flagellomere pale brown or apical 1/4 brown; remaining flagellum dark brown. First flagellomere 1.5–1.6× longer than second flagellomere (Fig. 2C).

**Figure 34.**
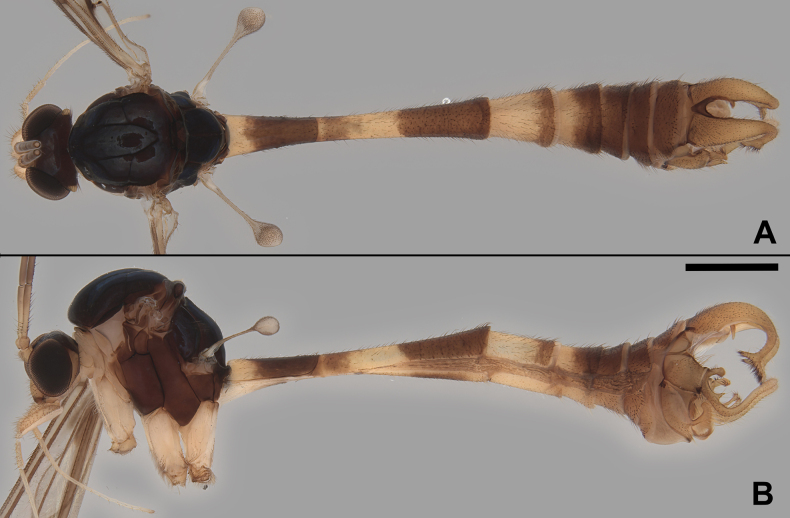
*Ptychoptera phutphi* Kolcsár & Fasbender, sp. nov. Male habitus. **A**. Dorsal; **B**. Lateral. Scale bar: 1 mm.

**Figure 35.**
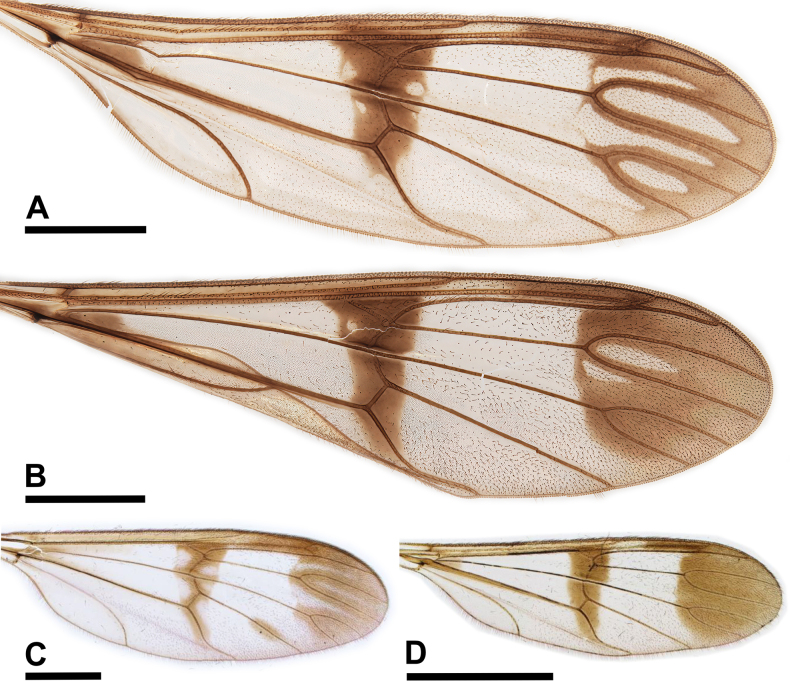
Wings. **A**. *Ptychoptera phutphi* Kolcsár & Fasbender, sp. nov. Paratype LPK0001; **B**. *Ptychoptera phutphi* Kolcsár & Fasbender, sp. nov. Paratype LPK0002; **C**. *Ptychoptera distincta* Brunetti, 1911 India: West Bengal State, Darjeeling. **D**. *Ptychoptera chalybeata* Alexander, 1956 Holotype. Scale bar: 0.5 mm.

***Thorax***. Antepronotum yellow; postpronotum yellow; prescutum uniformly shiny black, blue in certain angles; scutum uniformly shiny black, blue in certain angles; scutellum dark brown, with round brown spot in middle with dark brown setae; postalar callus dark brown; postalar wall dark brown; mediotergite uniformly shiny black, blue in certain angles; paratergite shiny black; propleuron yellow to pale brown; anepisternal cleft yellow; anepisternum dark brown; katepisternum dark brown; anterior basalare dark brown; posterior basalare dark brown; epimeron dark brown; laterotergite shiny black, blue in certain angles with patch of long setae; metanepisternum dark brown; metakatepisternum dark brown; metanotum dark brown (Figs 3B, 34). ***Wing***. Wing length/width ratio 3.5–3.6. Three brown pigment bands present: basal band from h to 1/3 of length R, between R and CuA; middle band present, around from fork of Rs to proximal angle of CuA, sometime not reaching pseudovein, cells occasionally with transparent patches (Fig. 3B vs. Fig. 35A, B); apical band reaching wing apex, extending from pterostigma past beyond M2, cell with (Fig. 35A, B) or without transparent patch (Fig. 3B); cells c pale brown, sc pale brown, transparent between apex of Sc and pterostigma, pterostigma brown. Membrane with microtrichia in every cells, less abundant in br, bm, cua, cup. Sc terminates 1.5× the length of r-m beyond fork of Rs; apex of R1 beyond fork of R_4+5_, Rs subequal to r-m, r-m joins R_4+5_ distal to R3 fork, R4 subequal to R_4+5_, proximal curve of R4 even arc. M_1+2_ fork 3/4 length of R_4+5_ fork. CuA sigmoidal distal to m-cu (Fig. 35A, B). ***Halter***. Stem basal 1/3 white or yellow, apical 2/3 pale brown; knob brown. Prehalter brown (Fig. 34). ***Legs***. Fore coxa yellow; fore trochanter pale brown; fore femur pale brown, apical 1/5 dark brown; fore tibia pale brown, apical 1/7 dark brown; fore tarsomere 1 brown; fore tarsomeres 2–5 dark brown; mid coxa yellow; mid trochanter pale brown; mid femur pale brown, apical 1/5 dark brown; mid tibia pale brown, apical 1/6 dark brown; mid tarsomere 1 brown; mid tarsomeres 2–5 dark brown; hind coxa yellow; hind trochanter pale brown; hind femur pale brown, apical 1/6 dark brown; hind tibia apical 1/7 dark brown; hind tarsomere 1 pale brown, apical 1/5 dark brown; hind tarsomeres 2–5 dark brown (Fig. 3B).

***Abdomen***. Tergite 1 anterior 1/3 yellow, posterior 2/3 dark brown; tergite 2 anterior 1/2 yellow with a basal pale brown patch, posterior 1/2 dark brown; tergite 3 yellow (Fig. 3B) or posterior margin brown (Fig. 34); tergite 4 anterior 1/2 yellow, posterior 1/2 brown; tergite 5 brown; tergite 6 brown; tergite 7 brown; sternite 1 yellow; sternite 2 anterior 1/2 yellow, posterior 1/2 pale brown; sternite 3 yellow, without accessory copulatory organ; sternite 4 pale brown; sternite 5 pale brown; sternite 6 pale brown; sternite 7 pale brown (Fig. 34).

**Figure 36.**
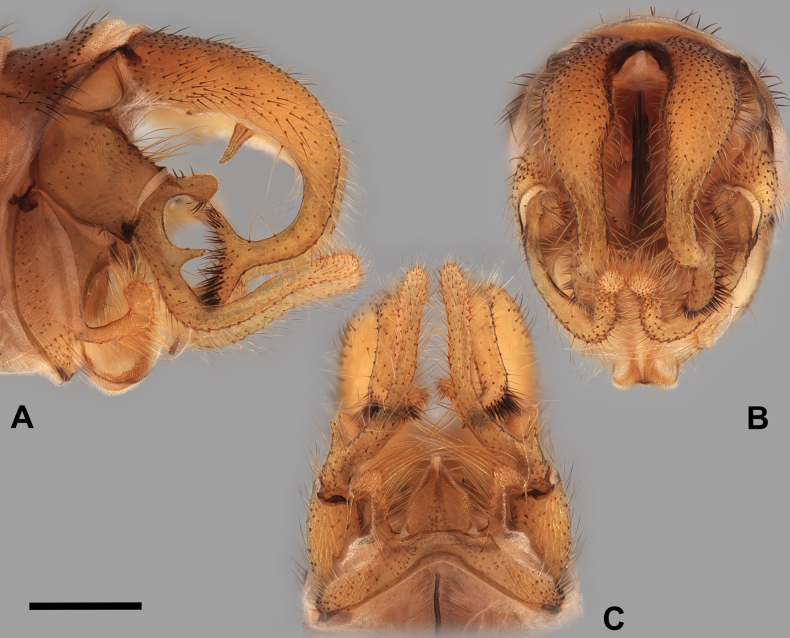
*Ptychoptera phutphi* Kolcsár & Fasbender, sp. nov. Unmacerated male terminalia. **A**. Lateral; **B**. Posterior; **C**. Ventral. Scale bar: 0.5 mm.

**Figure 37.**
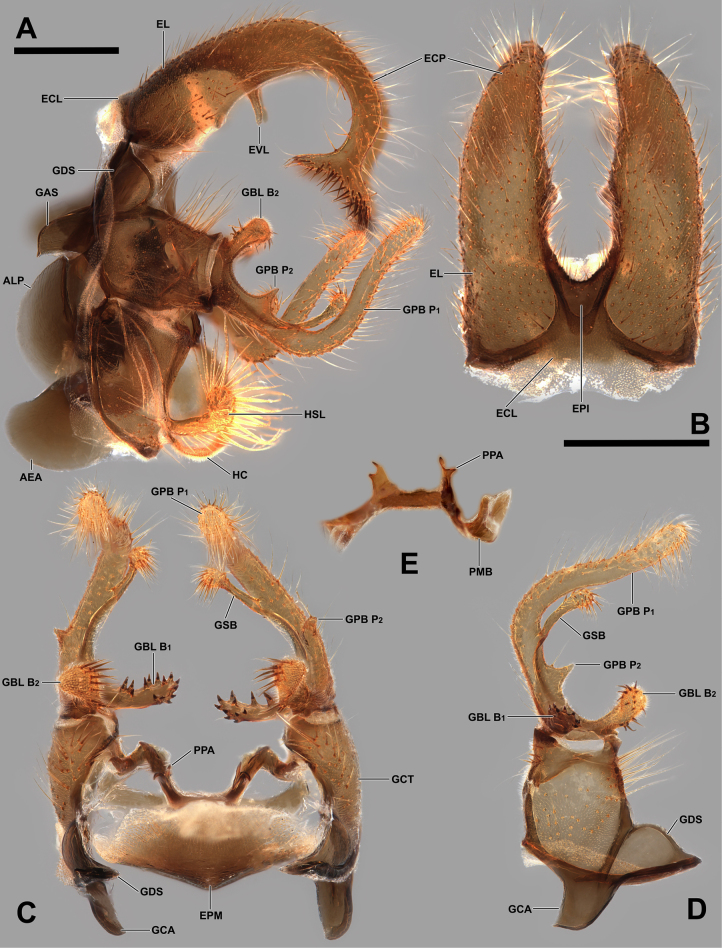
*Ptychoptera phutphi* Kolcsár & Fasbender, sp. nov. **A**. Overall, lateral; **B**. Epandrium, dorsal; **C**. Gonopods and parameres, dorsal; **D**. Gonopod, inner lateral; **E**. Parameres, posterior. Scale bars: 0.5 mm (**A**); 0.5 mm (**B–E**).

***Male terminalia***. Pale brown (Fig. 36). Epandrial collar complete. Epandrial apodemes well developed, thread-like, extending across gonocoxal apodeme to hypandrium, with desclerotized breaks present at epandrium and hypandrium (Fig. 37A). Epandrial lobes digitiform, elongate, robust (Figs 36A, 36B, 37A, 37B). Contiguous epandrial clasper indistinguishable from epandrial lobe, strongly curved anteroventrally; apex unequally bilobed, dorsal lobe shorter, both lobes covered with strong black setae (Figs 36A, 36B, 37A, 37B). Ventromesal lobe of epandrium present, finger-like, narrowing toward tip, directed ventrally (Figs 36A, 37A). Epiproct readily apparent, posterior margin of epiproct U-shaped, completely separating epandrial lobes (Fig. 37B). Hypoproct small sclerite, poorly distinguishable, fused with subepandrial sclerite, membranous (Figs 36B, 37B, 37C). Subepandrial membrane fully sclerotized, large; anterior-dorsal sclerite much larger than ventro-posterior sclerite; lateral sclerites separated from ventral sclerite (Fig. 37C). Parameral bridge undivided, rotated ventrally, approx. W-shaped (Fig. 37C); a small, pointed lobe present at the lateral corner near joint of gonocoxite, directed dorsally, visible only in posterior view (Fig. 37E). Apical process with two acute spines: inner spine directed apically, outer spine curved laterally (Fig. 37C, E). Gonocoxal apodeme triangular, rounded (Fig. 37D). Dorsal spur of gonocoxite present, very large, nearly equilateral, posteriorly rounded (Fig. 37A, D). Gonocoxite approx. pentagonal (Fig. 37A, D). Gonocoxal lobes absent; a few short black setae present on ventromedial margin between base of gonostylus and base of parameres (Fig. 37D). Basal lobe of gonostylus divided into two branches: posterior branch (B_1_) directed inward, approx. spoon-shaped, with 13–16 prominent, scattered, stout black spines on outgrowths (Fig. 37A, D); anterior-dorsal branch (B_2_) on dorsal side, triangular in dorsal view, triangular with narrow petiole in lateral view (Fig. 37A, D), covered with long setae.

**Figure 38.**
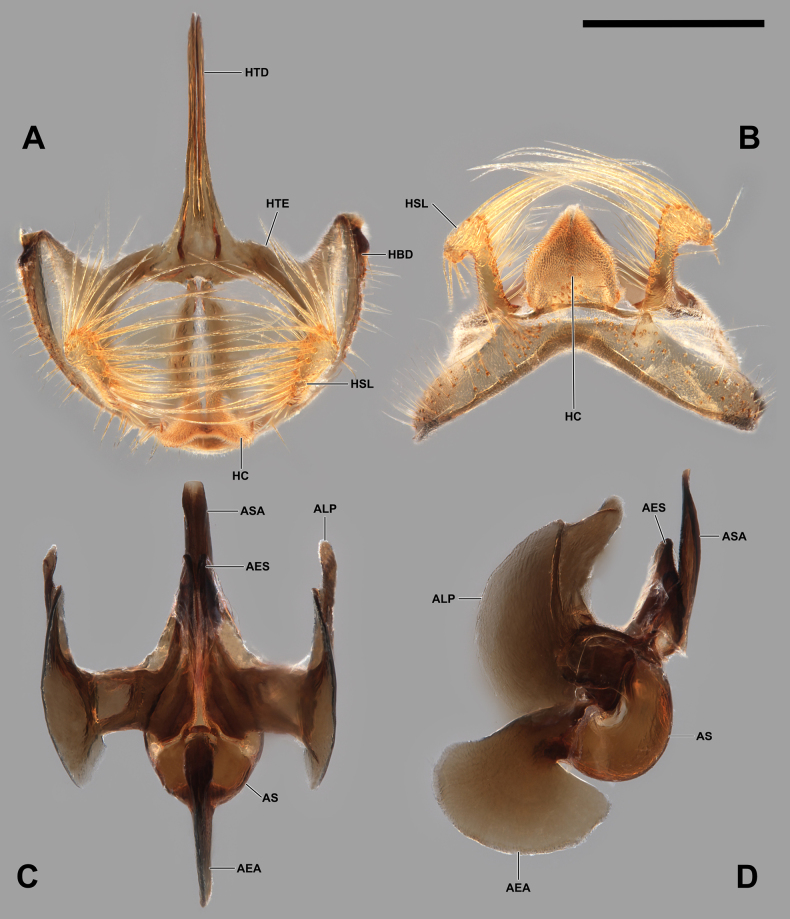
*Ptychoptera phutphi* Kolcsár & Fasbender, sp. nov. Male terminalia. **A**. Hypandrium, posterior; **B**. Hypandrium, ventral; **C**. Aedeagus, anterior; **D**. Aedeagus, lateral. Scale bar: 0.5 mm.

Primary branch (P_1_) long, finger-like, curved dorsally at a right angle at 1/2 its length, tip rounded, not dilated (Figs 36A, 37A, 37D); additional subdivision of primary branch (P_2_) on dorsal side, isosceles-triangular, pointed, directed dorsally (Fig. 37A, C, D). Secondary branch finger-like, directed dorsally, dilated apically, with dense patch of pale brown setae (Fig. 37A, C, D). Hypandrium with basal division band-like in lateral view (Figs 36A, 37A); basal scale absent; spathate lobe present very large, finger-like, apex dilated, directed posteriorly and curving dorsally at a perpendicular angle in lateral view (Figs 36A, 37A), covered with very long setae directed medially, setae greatly overlapping and completely covering hypandrial conjunctive (Fig. 38A, B). Hypandrial conjunctive present, reaching base of terminal division, triangular, ventrally about twice as wide as dorsally (Fig. 38A). Terminal division with well-developed lateral extensions connected to basal division (Fig. 38A). Apex triangular, ending in pair of mostly fused, very long needle-like lobes extending dorsoanteriorly, nearly the length of basal division (Fig. 38A). Aedeagus tilted 20 degrees from vertical in lateral view (Fig. 38D). Aedeagal sclerite tapering, apex slightly divergent in dorsal view (Fig. 38C), rounded apically in lateral view (Fig. 38D); subequal in length to sperm sac; apical apodemes present, originating distinctly proximal to sclerite apex. Lateral ejaculatory processes with distinct anterior angle, discoid apodeme elliptical ventrally; dorsal apex triangular; slightly larger than ejaculatory apodeme in lateral view (Fig. 38C). Sperm sac subspherical, slightly smaller than ejaculatory apodeme in lateral view (Fig. 38C). Ejaculatory apodeme fan shaped, curved ventrally (Fig. 38C), not adjacent to base of aedeagal sclerite, extending anteriorly into segment VII (Fig. 37A). Subaedeagal sclerite longer than aedeagal sclerite, parallel sided rod with squared tip in dorsal view (Fig. 38D). ventral arm of subaedeagal sclerite absent.

**Female**. Unknown.

##### Distribution.

Thailand (western Thailand).

##### Habitat.

The species was collected in a moist evergreen forest with a completely closed canopy, using a Malaise trap set beside a permanent stream ~ 2 m wide and 40 cm deep. In the same habitat, *Ptychoptera annandalei* and *P. perbona* were also collected.

##### Etymology.

The species epithet *phutphi* is the Thai word ภูตผี meaning ghost, referring to the common English name “phantom crane flies”. The epithet is a noun in apposition.

##### Remarks.

*Ptychoptera phutphi* is distinctive for its digitiform epandrial claspers with a T-shaped apex and the distal portion of the primary branch (P_1_) of the gonostylus being directed dorsally at nearly a right angle. The latter is found in no other *Ptychoptera*, and while there are some other digitiform species with a secondary spur or lobe on the distal portion of the epandrial clasper (*Ptychoptera sculleni* Alexander, 1943, *Ptychoptera yunnanica* Zhang & Kang, 2021) none are so extreme as this species. There is a ventrally directed spine on the ventromedial surface of the epandrial clasper, about 1/4 of the length from the epiproct towards the apex. The position to this lobe is similar to the flattened ventromesal lobe found in the *P. albimana*, *P. lenis* and *P. tibialis* groups. This spine is not nearly as developed as the flattened, leaflike structure provided with numerous sensilla found in those taxa, but the position suggests it may potentially be homologous. Based on the structure of the gonostylus, particularly the upturned apex, *Ptychoptera phutphi* does not currently fit into a recognized species group, although the elongate primary branch (P_1_) of the gonostylus and structure of the secondary branch are similar to *Ptychoptera javensis* Alexander, 1937, and *Ptychoptera bannaensis* Kang, Yao & Yang, 2013. Both of those species have modifications to the apex of the epandrial clasper (slightly spatulate in *P. javensis*, apical third thickened in *P. bannaensis*), of which the T-shaped lobe of *P. phutphi* may be an extreme example.

Two species in the Oriental region exhibit a similar wing pattern: *Ptychoptera chalybeata* Alexander, 1956, described from northern Thailand (“Huey Kao,” most likely referring to Huai Kaeo in Chiang Mai Province), and *P. distincta* Brunetti, 1911, described from West Bengal, India. *P. chalybeata* is currently known only from its male holotype. Its wing pattern closely resembles that of the *P. phutphi* holotype (Fig. 3B), showing three distinct dark bands, with the apical band reaching the wing margin and lacking transparent patches (Fig. 35D). However, the male terminalia of *P. chalybeata* are quite distinct from those of *P. phutphi* and more closely resemble those of *P. perbona*. *Ptychoptera distincta*, on the other hand, is known only from the female holotype and another female identified by C.P. Alexander as *P. distincta* from Arunachal Pradesh (Fig. 35C). Both share a similar wing pattern, with the middle and apical bands containing transparent patches (Fig. 35C), as seen in the paratypes of *P. phutphi* (Fig. 35A, B). However, the wing venation differs: in *P. distincta*, the fork of R_1_+_2_ lies at the same level as the fork of M_1_+_2_, whereas in *P. phutphi* it is situated beyond the fork of M_1_+_2_. Additionally, R_2_ is as long as R_1_ in *P. distincta*, but is almost indistinct in *P. phutphi*. The two species also differ in abdominal coloration; however, we consider this character less reliable for taxonomic differentiation, as both sexual dimorphism (see *P. hantu* and *P. annandalei*) and intraspecific variation occur among the species discussed in this paper.

#### Ptychoptera srilankaensis

Taxon classificationAnimaliaDipteraPtychopteridae

Paramonov sp. nov.

2D6B13BA-0359-5F06-AE17-30ACE51DB4D4

https://zoobank.org/424E2E35-163E-4EB9-88C4-D1F74473F15B

Figs 14A, 39–42

##### Type material.

***Holotype*. Sri Lanka** • male; Nuwara Eliya, 6.977°N, 80.756°E, 1990 m, 24.XII.2012, leg. N. Vikhrev; ZISP. HOLOTYPE *Ptychoptera srilankaensis* Paramonov, sp. nov. [red label].

##### Diagnosis.

Male terminalia with epandrial lobes subquadrate, epandrial clasper reduced to ovoid tubercle; gonocoxite with spike-like dorsal lobe; basal lobe of gonostylus with three spikes. Basal division of hypandrium with broad setose lobes on the posterior margin, covering basal portion of the terminal division.

##### Description.

**Male. *Measurements***: Wing length 8 mm, width 2 mm (*n* = 1). Antenna 5.7 mm.

***Head***. Vertex shining black, glabrous; frons extremely narrow strip dark brown; occiput black, stemmatic bulla is not observed; gena black; occiput without setae, gena with brown setae; facial sclerite small, dark brown, yellow below the scapes; clypeus rhomboidal, inflated, black, with few dark brown sensilla; palpus yellow, first segment pale brown; labrum narrowly spade or pentagonal shaped, anterior margin dark brown; labium pale brown; labellum pale brown with brown setae. Scape, pedicel yellow; flagellum brown, setae on antenna dark brown; first flagellomere 1.9× longer than second flagellomere (Fig. 14A).

**Figure 39.**
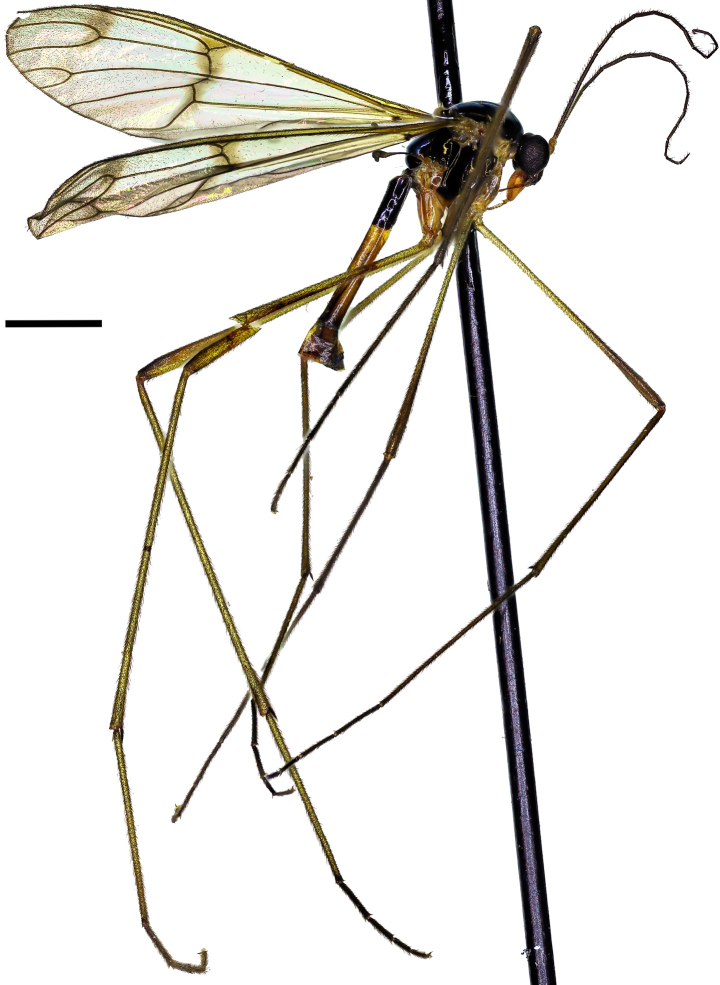
*Ptychoptera srilankaensis* Paramonov, sp. nov. Habitus. Scale bar: 2 mm.

***Thorax***. Antepronotum yellow; postpronotum yellow; prescutum uniformly shiny black, blue in certain angles; scutum uniformly shiny black, blue in certain angles; scutellum yellow-brown, with dark brown setae; postalar callus brown; postalar wall pale brown; mediotergite uniformly shiny black, blue in certain angles; paratergite black; propleuron yellow; anepisternal cleft yellow; anepisternum dark brown; katepisternum dark brown, anterior basalare dark brown; posterior basalare dark brown; epimeron dark brown; laterotergite dark brown with patch of long setae; metanepisternum yellow; metakatepisternum yellow; metanotum yellow (Fig. 39). ***Wing***. Wing length/width ratio 3.4. Two pigment bands present, basal band absent; middle band from Rs to medial curve of CuA, apical band from R_1+2_ fork to R_4+5_ fork, with spot at M fork. Membrane with macrotrichia in all cells except br, for proximal portions of bm. All veins covered in short stout macrotrichia. Sc terminates 2× the length of r-m, beyond fork of Rs. Rs equal r-m; r-m joins R_4+5_ distal to R_3_ fork; R_4+5_ stem slightly shorter then R_4_, proximal curve of R_4_ squared off. M_1+2_ fork ~ 1/2 as long as R_4+5_ fork. CuA sigmoidal distal to m-cu. ***Halter***. Stem basal 1/5 pale brown, apical 4/5 brown; knob brown. Prehalter pale brown (Fig. 39). ***Legs***. Fore coxa yellow; fore trochanter yellow; fore femur pale yellow, apical 1/2 brown; fore tibia brown; fore tarsomere 1 pale brown, mid tarsomeres 2–5 dark brown; mid coxa yellow; mid trochanter yellow; mid femur pale yellow, apical 1/2 dark brown; mid tibia pale brown; mid tarsomere 1 pale brown, apical 1/2 dark brown; mid tarsomeres 2–5 dark brown; hind coxa yellow; hind trochanter pale brown; hind femur basal 1/3–1/2 brown, middle pale brown, apical 1/6 dark brown; hind tibia basal 1/5 brown, apical 4/5 pale brown; hind tarsomere 1 pale brown, apical 1/5 dark brown; hind tarsomeres 2–5 dark brown (Fig. 39).

***Abdomen***. Tergite 1 black; tergite 2 anterior yellow, 2/3 yellow, posterior 1/3 brown; tergite 3 anterior brownish black (then dissected); sternite 1 black; sternite 2 anterior 2/3 yellow, posterior 1/3 brown; sternite 3 anterior brownish black; without accessory copulatory organ. Rest of abdomen dissected (Fig. 39).

***Male terminalia***. Brown, epandrium more darkened than rest of terminalia. Epandrial collar complete. Epandrial apodemes well developed, thread-like across gonocoxal apodeme to hypandrium, breaks present at epandrium and hypandrium. Epandrial lobes subquadrate, posterior margin slanted anteromedially (Fig. 41A, B). Epandrial clasper reduced to ovoid tubercle on posterior surface, well sclerotized, covered by strong black spikes (Fig. 41A, B). Ventral surface of epandrial lobe with circular knob covered by strong black spikes (Fig. 41B). Epiproct readily apparent, reaching posterior margin of epandrium; posterior margin of epiproct U-shaped, completely separating epandrial lobes (Fig. 41A). Hypoproct missing on holotype. Parameral bridge complete, narrowly attached to gonocoxite (Fig. 41C, D); apical process of paramere triangular, apex directed posterolaterally, extending to basal lobe of gonocoxite (Fig. 41C–F). Gonocoxal apodeme acutely pointed, 1/4 length of gonocoxite. Dorsal spur present, short, rounded (Fig. 41C–E). Gonocoxite approx. rectangular. Dorsal gonocoxal lobe present, heavy sclerotized, elongate spike directed medially (Fig. 41C–E). Gonostylus with basal lobe divided into two branches: posterior branch (B_1_) subdivided into two heavily sclerotized, elongate spikes, directed posteriorly; anterior branch (B_2_) heavy sclerotized, elongate spike, directed dorsally (Fig. 41C–E).

**Figure 40.**
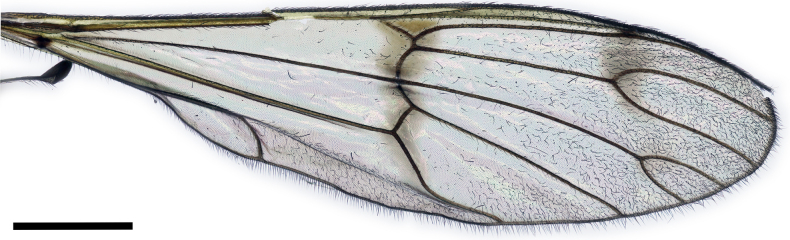
*Ptychoptera srilankaensis* Paramonov, sp. nov. Wing. Scale bar: 0.5 mm.

**Figure 41.**
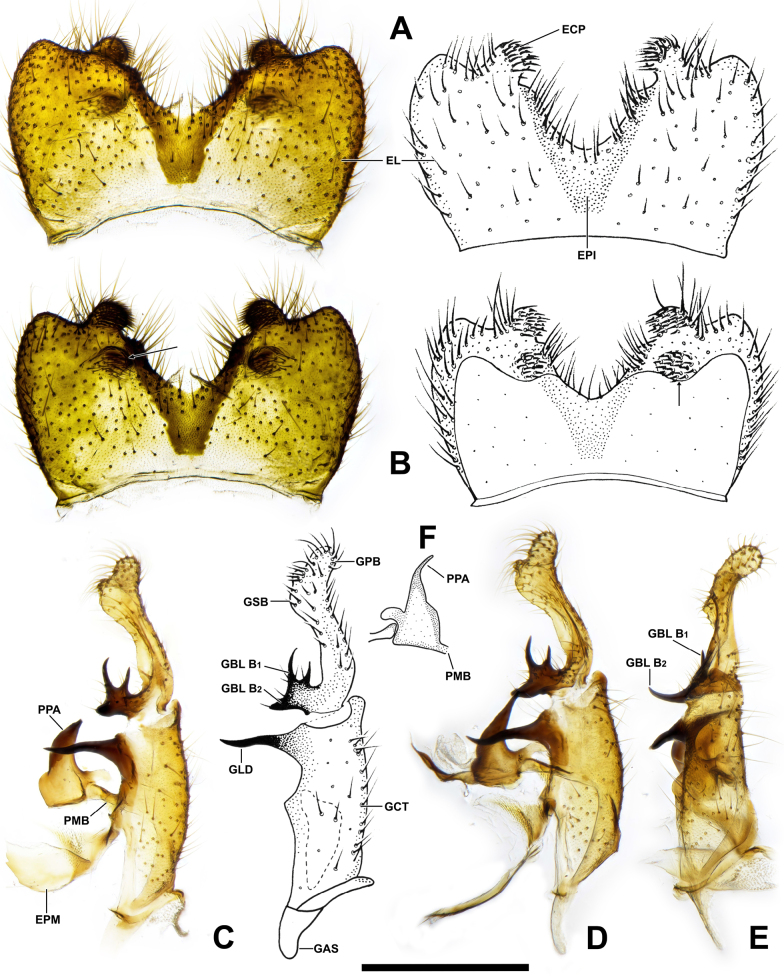
*Ptychoptera srilankaensis* Paramonov, sp. nov. **A**. Epandrium, dorsal; **B**. Epandrium, ventral; **C**. Gonopod and paramere, dorsal; **D**. Gonopod and paramere, ventral; **E**. Gonopod, outer lateral; **F**. Paramere. Scale bar: 0.5 mm.

**Figure 42.**
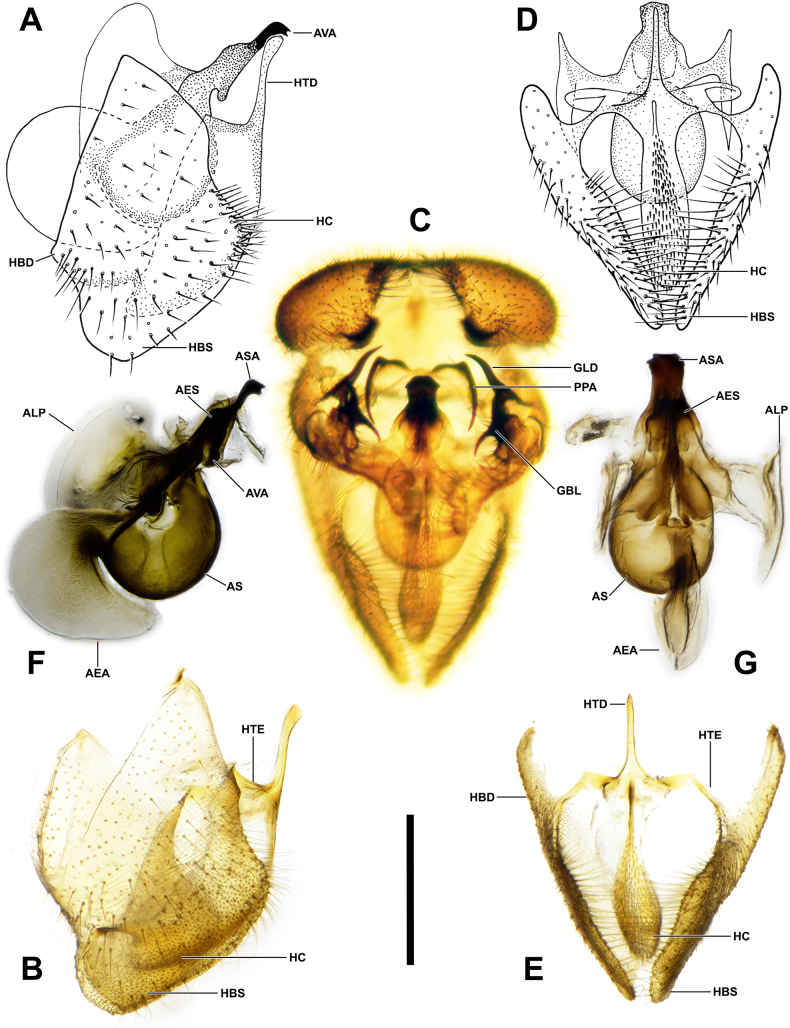
*Ptychoptera srilankaensis* Paramonov, sp. nov. **A**. Hypandrium and aedeagus, lateral; **B**. Hypandrium, lateral; **C**. Overall, posterior; **D**. Hypandrium and aedeagus, posterior; **E**. Hypandrium, posterior; **F**. Aedeagus, lateral; **G**. Aedeagus, anterior (slightly rotated). Scale bar: 0.5 mm.

Apical lobe finger-like, with a low ridge on the inner-dorsal surface, weakly laterally compressed, slightly curved ventrally. Primary and secondary branches weakly separated, secondary branch rounded medially with a few setae on its outgrowth (Fig. 30). Basal division of hypandrium subquadrate not well delineated from broad posteroventral lobes (?spathate lobes), lobes broadly rectangular laterally (Fig. 42A, B), covered by long setae, directed medially, covering hypandrial conjunctive (Fig. 42C–E). Hypandrial conjunctive present, elongate drop-shaped, covered by short setae. Terminal division with well-developed lateral extensions connected to basal division, break in almost right angle 1/2 of length (Fig. 42D, E). Apex triangular, ending in a very long needle-like lobe in posterior view (Fig. 42D, E), tip slightly widens and curving posteriorly in lateral view (Fig. 42A, B). Aedeagal sclerite with apical apodeme (Fig. 42G). Lateral ejaculatory processes with discoid apodeme scallop shaped, dorsal portion subequal to ventral portion, subequal to ejaculatory apodeme in lateral view (Fig. 42F). Sperm sac ovoid, subequal in length to ejaculatory apodeme in lateral view (Fig. 42F). Ejaculatory apodeme bluntly claw-shaped, closely following anterior margin of sperm sac, apex tapering (Fig. 42F). Subaedeagal sclerite quadrate, ventrally with two teeth at apex (Fig. 42F, G), resting at the apex of needle-like lobe of terminal division (Fig. 42A).

**Female**. Unknown.

##### Distribution.

Sri Lanka.

##### Etymology.

The species epithet is given in honor of the English name of the country Sri Lanka. The epithet is to be treated as an adjective in apposition.

##### Remarks.

*Ptychoptera srilankaensis* is challenging to place within the wider morphological context of the genus. The gonostylus is similar to *P. perbona* in having weakly separated primary and secondary branches of apical lobe, but the structure of the gonocoxite, epandrium and hypandrial conjunctive are quite different. The epandrium is strikingly similar to that found in the *P. pendula* group of North America with the reduction of the epandrial clasper, which is reduced to the knob-like structure on the posterior margin of the epandrial lobe. The gonostylus basal lobe with its elongate darkened spines is also similar to the *P. pendula* group. Perhaps most intriguing is the structure of the basal division of the hypandrium. There is a pair of broad setose lobes on the posterior margin, obscuring much of the basal portion of the terminal division. While their base is poorly differentiated from the main body of the basal division their position suggests they may be homologous with the articulated spathate lobes seen in many *Ptychoptera* species.

## Discussion

During the past two decades, 21 new species of *Ptychoptera* have been described from the Oriental (10 species) and Eastern Palaearctic (11 species) regions, representing an 84% increase in the known diversity of the genus in Asia. This substantial growth in species numbers underscores the high diversity of the Asian *Ptychoptera* fauna. In the present study, we describe five additional species and synonymize two, thereby increasing the number of recognized Oriental species to 29 and the global total to 95. The present contribution constitutes the first step toward a comprehensive revision of the Southeast Asian Ptychopteridae.

Most of the Oriental *Ptychoptera* species are known solely from their type series, making the relative endemism of species in the region unclear. New records provide information on previously described species, including our first understanding how far individual *Ptychoptera* species range in southeast Asia. Based on our new records it appears that both *P. annandalei* and *P. perbona* are associated with the Northern Indochina Subtropical Forest ecoregion. Still, this is based on extremely limited locality data and additional collecting is warranted to uncover the true extent of both these species range, to say nothing of the numerous others still known only from their type localities.

The Oriental *Ptychoptera* fauna have not had their morphology or relationships thoroughly examined, and it is difficult to place species from the region in a wider context. Although Fasbender (2014) provided redescriptions of most Oriental *Ptychoptera*, they were based primarily on fragmentary slide mounted material from the C. P. Alexander collection. The complex and three-dimensional male genitalia were flattened and crushed during the mounting process, and important structures like the aedeagus and hypandrium were often not included on these slides by C. P. Alexander. This has made comparison with unflattened male genitalia difficult, which may lead to misinterpretations as demonstrated in case of *P. annandalei* and *P. cordata* syn. nov., and the subspecies of *P. perbona*.

Fasbender (2014) placed most of the Holarctic *Ptychoptera* species into species groups, but was only confident in placing three species from the Oriental Region into a species group: the *Ptychoptera tibialis* group including *Ptychoptera tibialis* Brunetti, 1911, *Ptychoptera garhwalensis* Alexander, 1959 and *Ptychoptera sikkimensis* Alexander, 1965 (with the subsequently described *Ptychoptera separata* Kang, Xue & Zhang, 2019). Fasbender tentatively suggested that *P. formosensis*, *P. persimilis*, and the species described here as *P. pseudosimilis* may represent a clade, but the limited material available at the time led him to refrain from formally placing those taxa. Additional species described from China since 2013 (Kang et al. 2013, 2019; Shao and Kang 2021; Zhang and Kang 2021) with the material examined here have provided additional information and support for a *P. formosensis* group consisting of *P. formosensis*, *P. annandalei*, *P. emeica*, *P. lii*, *P. lushuiensis*, *P. persimilis*, *P. pseudosimilis*, *P. tianmushana*, *P. vietnamensis*, and *P. yunnanica*.

The *P. formosensis* group can be diagnosed by the digitiform epandrium, presence of spathate lobes of the hypandrium, lack of gonocoxal lobes, crescent shaped basal lobe of the gonostylus, and the primary branch of the gonostylus being fingerlike, with a large secondary branch that is flag-like anteriorly but tapers to a digitiform stylus provided with numerous black conical sensilla on the medial surface. The terminal apices of the secondary and primary branches are subequal in length, and usually crossed at the apex, giving the distal end of the gonostylus a bifid appearance. The configuration of the basal lobe, primary and secondary branches are all synapomorphies for the species group. To date the *P. formosensis* group is only recorded from the Oriental region and adjacent areas of Palaearctic China. These species can be challenging to differentiate from each other, as they share many features of body coloration, wing pigmentation/venation and male genital features. We have constructed a key to separate them, though due to the fragmentary material available of *P. persimilis* and *P. pseudosimilis* we had to focus primarily on the use of male genital characters.

In constructing the key we noted that *P. emeica* and *P. lii* could be separated solely on minor differences in wing pigmentation, which appear to be within the intraspecific variation seen in some other species. The figures of the male genitalia of *P. lii* (Kang et al. 2013) and *P. emeica* (Kang et al. 2019) show some subtle differences, though it is difficult to interpret whether these are artifacts as the illustration of *P. lii* is less detailed than the later *P. emeica*. As we have not examined specimens of either species, we tentatively treat them as distinct species for the time being. There is also considerable similarity between *P. persimilis* and *P. lushuiensis*. There are a few genital differences as noted in our key, but these may be artifacts of the slide mount of the *P. persimilis* holotype. When additional material of *P. persimilis* becomes available it should be compared with *P. lushuiensis* to determine if they actually represent separate species.

Our understanding of the Oriental *Ptychoptera* is still in flux, and one of the goals of future descriptive work should be construction of a comprehensive key to the regional fauna. For species not covered in our *P. formosensis* group key we recommend consulting Fasbender’s (2014) key to the *Ptychoptera* examined in his study and the Kang at al. (2022) key to the Chinese species for the time being. We consider the development of an online catalogue and database for the Ptychopteridae of the world, including both extinct and extant species, to be of great importance at this stage.

## Supplementary Material

XML Treatment for Ptychoptera annandalei

XML Treatment for Ptychoptera persimilis

XML Treatment for Ptychoptera pseudosimilis

XML Treatment for Ptychoptera vietnamensis

XML Treatment for Ptychoptera hantu

XML Treatment for Ptychoptera perbona

XML Treatment for Ptychoptera phutphi

XML Treatment for Ptychoptera srilankaensis

## Additional information

### Artificial Intelligence (AI) use

Regarding the use of AI in the preparation of this manuscript, the authors declare the following:

AI-assisted tools were used exclusively for proofreading, improving the English language, and formatting the citations; no scientific content was generated by AI.

### Author ORCIDs

L.-P. Kolcsár https://orcid.org/0000-0001-7784-2386

N. Paramonov https://orcid.org/0000-0002-3497-9785

W. Srisuka https://orcid.org/0000-0002-1868-0601

Y. Ang https://orcid.org/0000-0001-5889-018X

### Data availability

All of the data that support the findings of this study are available in the main text.
